# Anticancer Activity of Region B Capsaicin Analogs

**DOI:** 10.1021/acs.jmedchem.2c01594

**Published:** 2023-03-31

**Authors:** Kathleen C. Brown, Kushal J. Modi, Reagan S. Light, Ashley J. Cox, Timothy E. Long, Rama S. Gadepalli, John M. Rimoldi, Sarah L. Miles, Gary Rankin, Monica Valentovic, Krista L. Denning, Maria T. Tirona, Paul T. Finch, Joshua A. Hess, Piyali Dasgupta

**Affiliations:** † Department of Biomedical Sciences, Toxicology Research Cluster, Joan C. Edwards School of Medicine, Marshall University, 1700 Third Avenue, Huntington, West Virginia 25755, United States; ‡ Department of Pharmaceutical Sciences and Research, Marshall University School of Pharmacy, 1538 Charleston Ave, Huntington, West Virginia 25701, United States; § Department of Biomolecular Sciences, School of Pharmacy, Thad Cochran Research Center, University of Mississippi, University Avenue, University, Mississippi 38677, United States; ∥ Department of Pathology, Joan C. Edwards School of Medicine, Marshall University, 1400 Hal Greer Boulevard, Huntington, West Virginia 25755, United States; ⊥ Department of Hematology-Oncology, Edwards Cancer Center, Joan C. Edwards School of Medicine, Marshall University, 1400 Hal Greer Boulevard, Huntington, West Virginia 25755, United States; # Department of Oncology, Edwards Cancer Center, Joan C. Edwards School of Medicine, Marshall University, 1400 Hal Greer Boulevard, Huntington, West Virginia 25755, United States

## Abstract

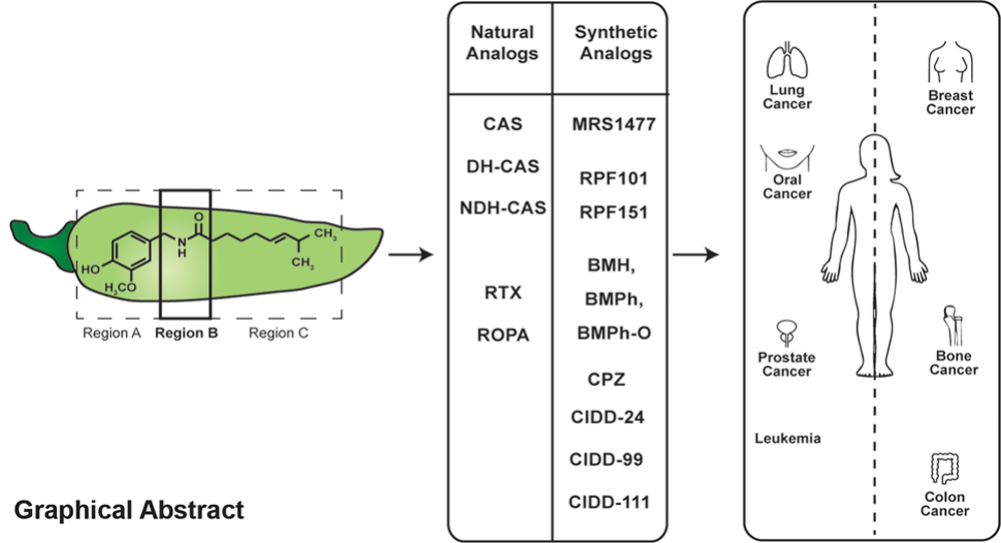

The heterocyclic vanilloid compound capsaicin is responsible for the spicy and pungent flavor of chili peppers. Several convergent studies have shown that capsaicin suppresses the growth of multiple human cancers. Apart from capsaicin, natural and synthetic capsaicin-like compounds display growth suppressive activity in human cancers. The pharmacophore of capsaicin is comprised of three regions, namely region A (the aromatic ring), region B (the amide bond), and region C (the side chain). The present manuscript describes the isolation and synthesis of capsaicin analogs which have structural modifications in region B of the molecule. Furthermore, the pharmacokinetic properties, anticancer activity of region B capsaicin analogs, as well as the signaling pathways (underlying the growth-inhibitory effects of region B capsaicin analogs) have also been described. The discovery of novel, second-generation region B capsaicin analogs may foster the hope of innovative nutrition-based combination therapies in human cancers.

## 1 Introduction

Capsaicinoids are a class of alkaloid compounds that are responsible for the hot, spicy, and pungent taste of chili peppers.^1^ The best known capsaicinoid is “capsaicin” which is isolated from the chili peppers of the genus *Capsum*. Capsaicin (**1**) is a colorless, odorless alkaloid that is a solid with a melting point of 62 °C and a molecular formula of C_18_H_27_NO_3_. It is a lipophilic compound with solubility in organic solvents, alcohol, and oils.^2−4^ Several convergent studies have revealed **1** to be a potent pain-relieving agent that is incorporated in several over-the-counter analgesic creams and lotions.^2−4^ The pain-relieving activity of **1** is mediated by the transient receptor potential vanilloid (TRPV) receptor superfamily of ion-channel receptors on target cells. The TRPV family of receptors is comprised of six receptor subtypes designated TRPV1–6.^5,6^ Capsaicin (**1**) is a potent agonist of the TRPV1 receptor.^7^ The binding of **1** to TRPV1 results in a robust increase in intracellular calcium levels, leading to the eventual downregulation of substance P, a neuropeptide involved in the nociceptive signals from nerve endings to the brain and the release of inflammatory cytokines.^8−10^ These molecular events lead to “defunctionalization” of nociceptor fibers and ablation of pain sensation.^9^ The long-acting formulation of **1**, a transdermal patch marketed as Qutenza, is used in the clinic to relieve diabetic nerve pain and neuropathic pain associated with postherpetic neuralgia.^11,12^ Qutenza is an extended release, localized dermal delivery system containing 8% **1** (40 μg of **1** per cm^2^ of patch) and provides up to three months of pain relief for patients.^13^

**Figure 1:**
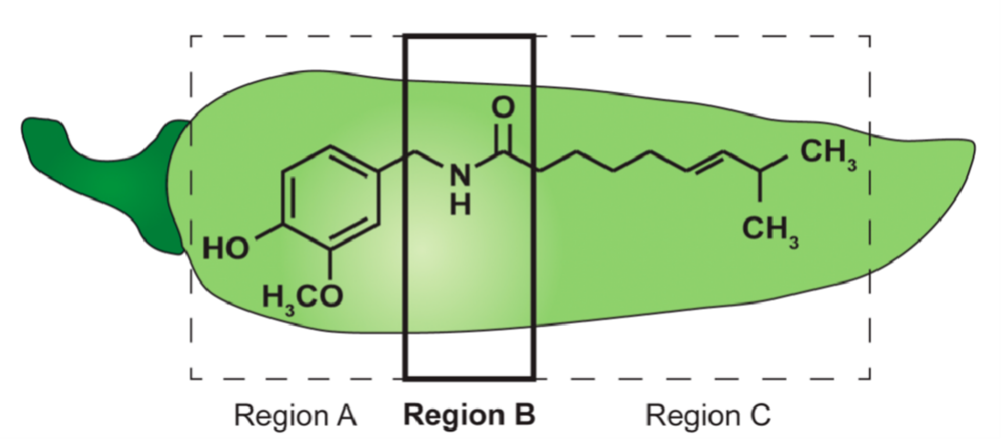
Structure of capsaicin with regions A, B, and C depicted.

Apart from its pain-relieving activity, **1** exerts a wide variety of biological and pharmacological activities which may have important applications in the therapy of human diseases.^14−16^ Published data reveal that **1** and other capsaicinoids display strong antioxidant properties, promote energy metabolism, suppress fat accumulation, combat inflammation,^17−19^ and suppress the growth and progression of a diverse array of human cancers. The antineoplastic activity of **1** has been observed in leukemia, breast, lung, prostate, brain, gastrointestinal, and gynecological cancers.^20−22^ In several types of human cancers, capsaicin inhibits tumor invasion and metastasis to distant organs.^23,24^ Apart from exerting direct growth-inhibitory activity toward human tumors, **1** sensitizes human cancer cells to the growth-suppressive effects of established chemotherapy drugs and radiotherapy.^25−31^ The multiple pharmacological activities of capsaicinoids have laid the foundation for detailed structure–activity-studies (SAR) focused on the development of potent, long-acting agonists and antagonists of the TRPV1 receptor with improved biological activity. Optical crystallography experiments conducted by Nelson and Dawson in 1923 deciphered the structure of **1**(32) (Figure 1). Pharmacological studies have divided the structure of **1** into three domains, namely region A, comprising of the aromatic ring, region B, comprising of the amide bond, and region C, comprising of the hydrophobic side chain.^4,33^

A variety of experimental techniques, including site-directed mutagenesis, patch-clamp recording, X-ray crystallography, cryo-electron microscopy, computational docking, and molecular dynamic simulation, have been used to delineate the interactions between **1** and its cognate receptor TRPV1.^34,35^ These studies have revealed that the phenolic ring and the chemical groups located in the meta and para positions in region A are vital for the analgesic activity of **1**. Any variation in the molecular structure which blocks the para hydroxyl group leads to a loss of TRPV1 binding and concomitant loss of biological activity.^36^ Consequently, it is not surprising that only a few analogs of **1** with modifications in region A have been reported in the literature and show diminished binding to TRPV1 and decreased pain-relieving activity compared to the parent compound.^33^ The greatest number of capsaicin analogs have been synthesized by structural modifications in region C. A prominent class of region C analogs are the *N*-acylvanillamides (N-AVAMs) which are nonpungent and display improved pain-relieving properties compared to **1**.^33,37^ Several published research papers have reported on the *in vivo* analgesic activity of natural and synthetic capsaicin analogs with structural modifications in region B.^33,38,39^ However, only a few studies have examined the anticancer activity of these region B capsaicin analogs. The primary goal of the present perspective article is to describe the isolation from natural sources, synthesis strategies, pharmacokinetics, and anticancer activity of region B-capsaicin analogs. Although, many region B capsaicin analogs have been reported in the literature, we will only discuss those capsaicin-mimetics that have been explored for their anticancer activity. Finally, we will discuss the signaling pathways underlying the anticancer activity of these region B capsaicin analogs. We believe that a detailed discussion of the anticancer activity of region B capsaicin analogs will be timely and relevant for researchers working in the field of drug discovery, drug delivery, and cancer therapeutics.

## 2 Natural and Synthetic Region B Capsaicin Analogs

**Figure 2:**
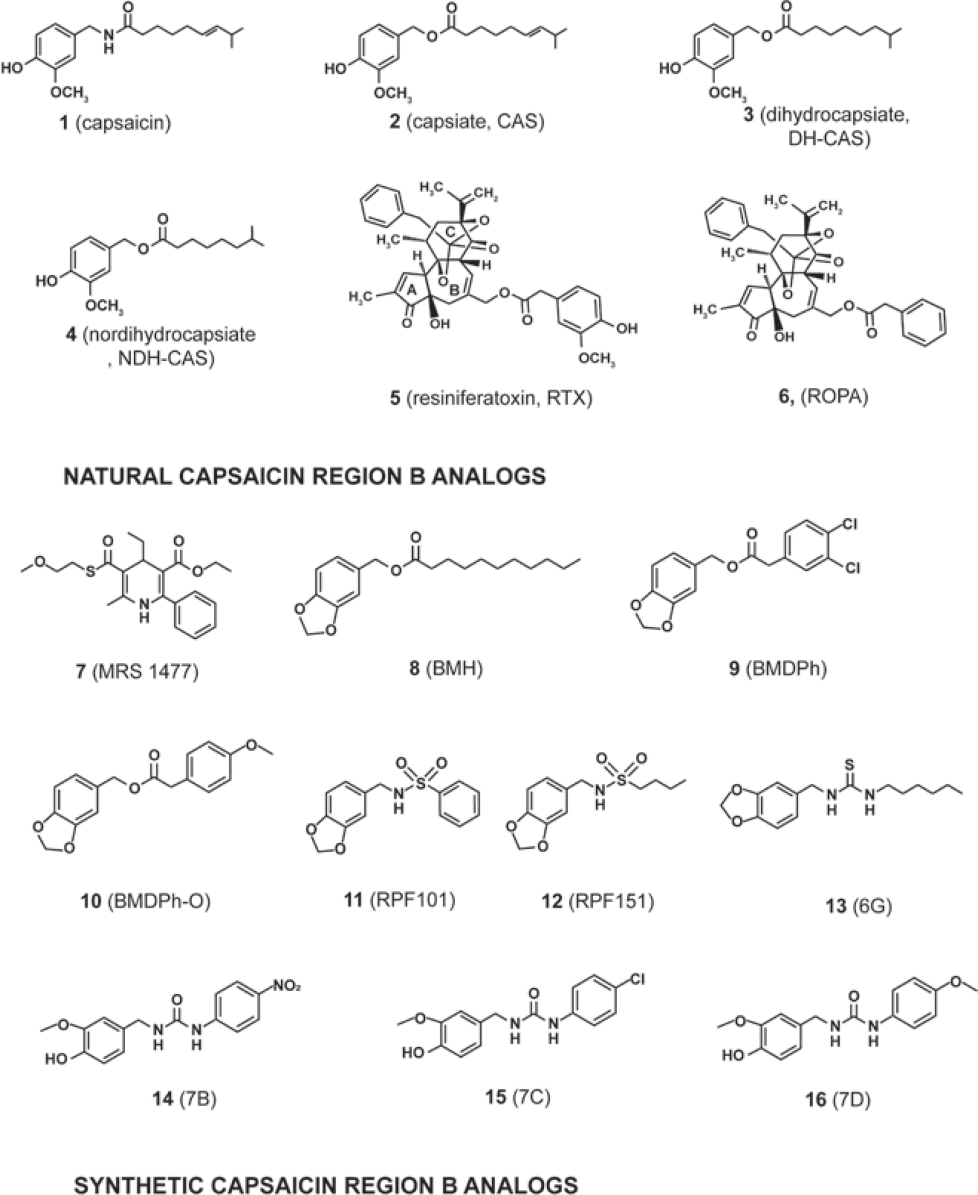
Natural and synthetic region B capsaicin analogs.

**Figure 3:**
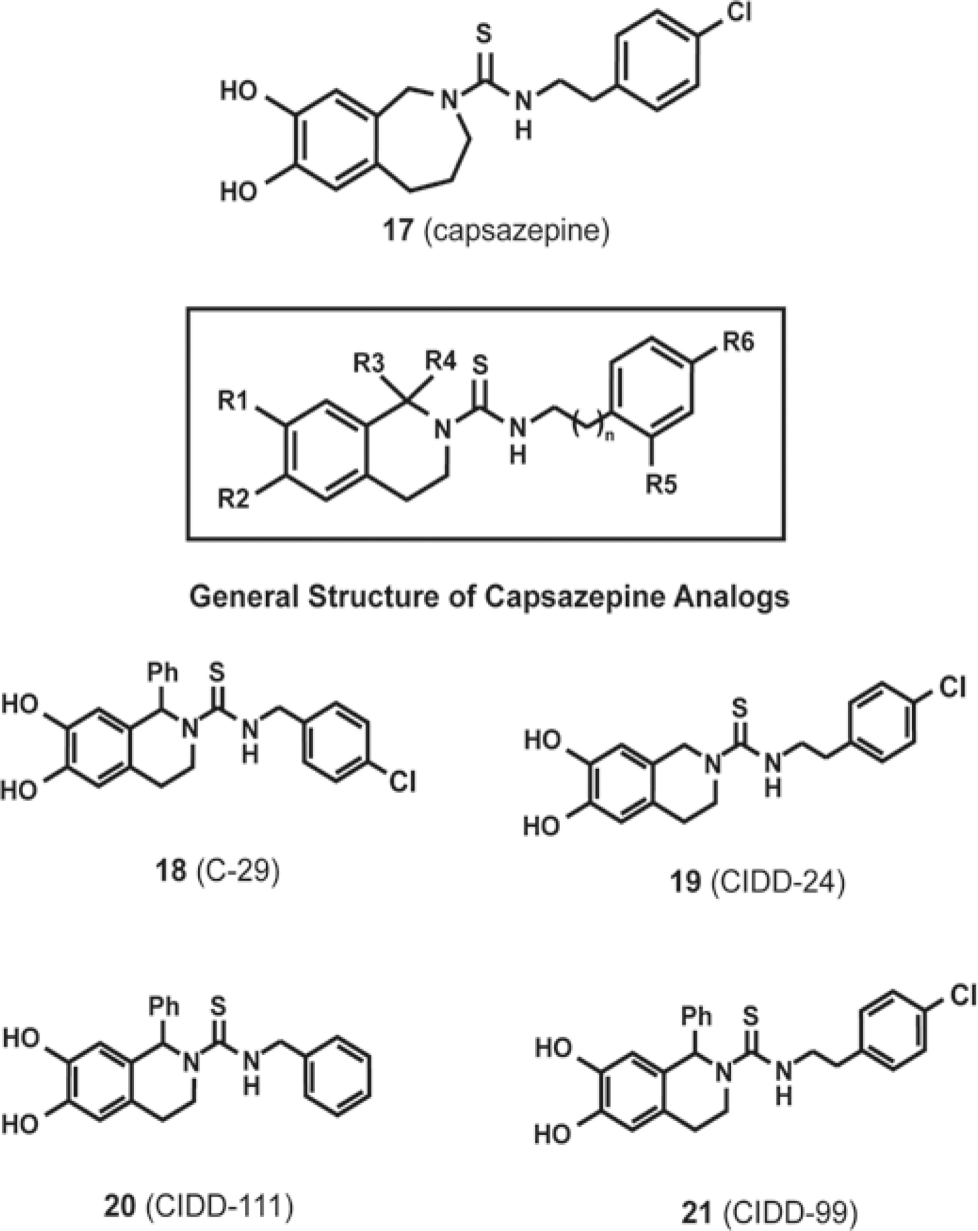
Region B capsaicin analogs based on the structure of **17**.

The present perspective article will discuss the growth-suppressive activity associated with the region B capsaicin analogs represented in Figures 2 and 3. Capsaicin (**1**) is the hot and spicy ingredient of chili peppers, and the antineoplastic activity of these analogs has been investigated *in vitro* and *in vivo*. Among these compounds, capsiate (**2**, CAS), dihydrocapsiate (**3**, DH-CAS), nordihydrocapsiate (**4**, NDH-CAS), resiniferatoxin (**5**, RTX), and resiniferonol 9,13,14-orthophenylacetate (**6**, ROPA) are isolated from natural sources. The remaining compounds have been generated by chemical synthesis. The structure of **2** is identical to **1** with the exception that **2** presents an ester moiety in region B while **1** contains an amide group. In addition to structural alterations to region B, all of the other compounds **3–21** have additional chemical modifications in region A and region C of their structures which have been explored as part of SAR studies.

In addition to **1**, the structure of the high affinity TRPV1 antagonist capsazepine (**17**) has been used as a scaffold to synthesize region B analogs with robust anticancer activity^33^ (Figure 3).

## 3 Isolation of Region B Capsaicin Analogs from Natural Sources

### 3.1 Isolation of Capsinoids from Chili Peppers

**Figure 4:**
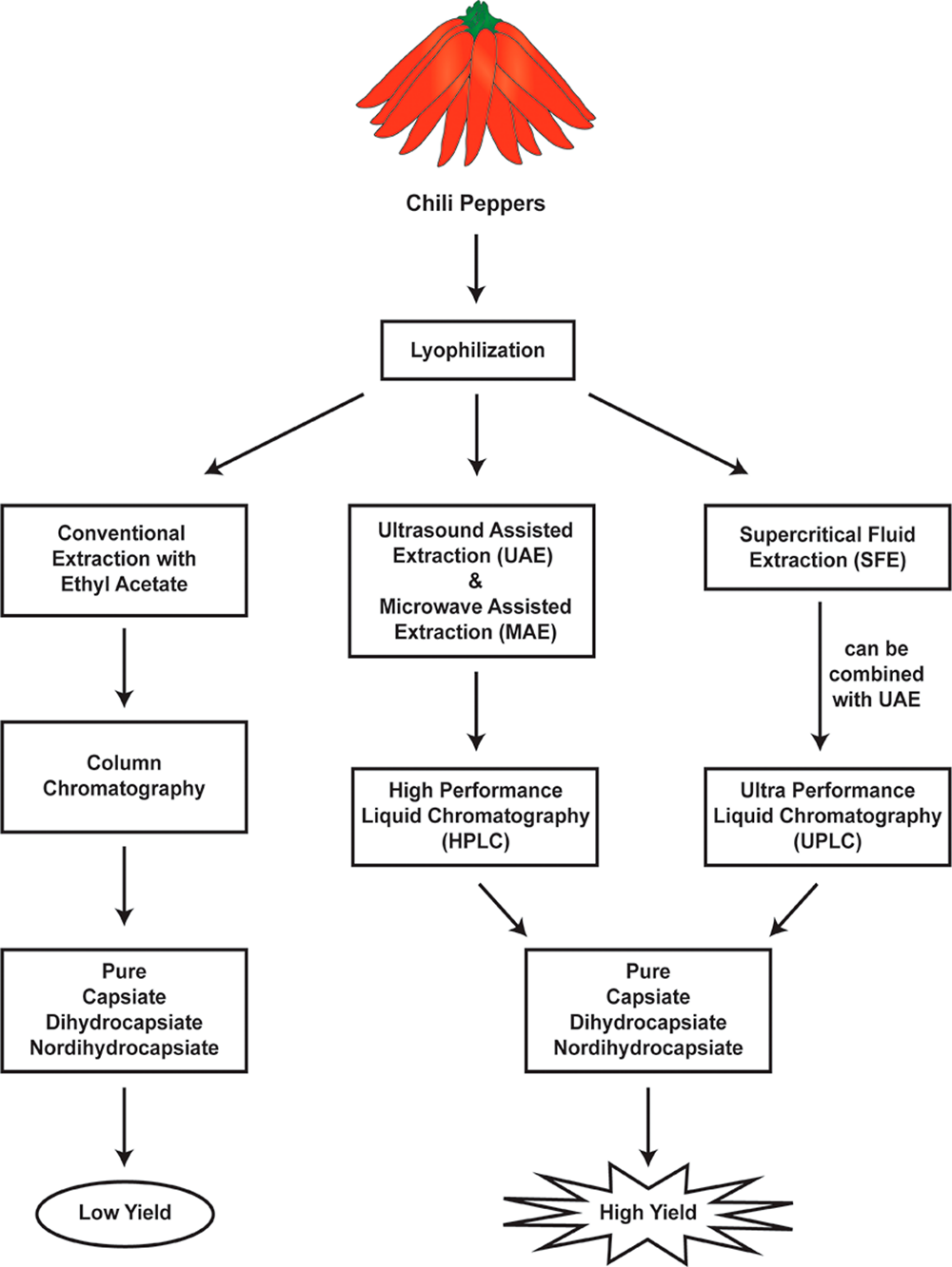
Extraction of region B capsaicin analogs from plants.

The capsaicin analogs **2**–**4** are collectively called “capsinoids” (Figure 2) and early research papers isolated these from the fruit of the CH-19 Japanese sweet pepper. The currently known extraction methods to obtain capsinoids from diverse strains of chili peppers are summarized in Figure 4. Almost all methods to isolate **2**–**4** used freeze-dried CH-19 peppers as the starting material.^40^ The lyophilized residue was homogenized and extracted three times with EtOAc, and the extract evaporated under reduced pressure to yield a product known as “oleoresin” which was chromatographed on a silica gel column to obtain a mixture of capsinoids. The eluate was rechromatographed using a reversed phase silica gel column and eluted with a 75% MeOH containing 0.05 M AgNO_3_ to obtain purified **2** and **3**.^40^ Apart from CH-19 peppers, capsinoids have also been isolated from many strains of chili peppers and from the peduncle and calyx parts of peppers, which are considered to be waste products.^1,41−43^ The germplasm 509-45-1 of the *Capsicum annum* pepper strain has been found to contain the highest amounts of capsinoids.^44^ The industrial large-scale extraction of **2** and **3** is performed using the fruit of 509-45-1 peppers.^45^ The fruit from the 509-45-1 pepper is ground to a fine powder and extracted three times with pentane over a period of 24 h, the extracts are filtered and subjected to liquid–liquid partitioning in a separating funnel using CH_3_CN.^46^ The CH_3_CN extract is then purified by HP20ss resin column chromatography to yield pure **2** and **3**.^46^ The HP20ss columns are made of macroporous polymeric bead-type resins designed to capture hydrophobic moieties from natural extracts.^47,48^ Its wide pore polymeric structure provides excellent broad-spectrum adsorption characteristics to purify a wide variety of bioactive compounds.

After **2** and **3**, the third most important capsinoid in CH-19 sweet peppers is **4**, although it is of relatively low abundance. The ratio of **2**, **3**, and **4** in CH-19 sweet peppers is about 5:3:1.^49^ Traditional reversed-phase HPLC methods usually result in the coelution of **3** and **4**. To overcome this technical challenge, ultrahigh performance liquid chromatography (UHP-HPLC) using a phenyl-hexyl column was used to purify **2**, **3**, and **4** from tabasco peppers.^50^ The phenyl-hexyl column is a mixed mode stationary phase in which the phenyl groups provide π–π interactions for compound association and the hexyl group offers additional hydrophobic interactions. The column was held at 55 °C to separate **2**, **3**, and **4**. A binary mobile phase of 0.2% HCO_2_H in H_2_O and MeOH enabled all three capsinoids to be eluted within 5 min of runtime.^50^ It was found that **2** was the most abundant capsinoid in tabasco peppers (838.4 ± 45 μg/g pepper), followed by **3** (372.8 ± 21 μg/g pepper), while **4** was the rarest capsinoid (122.8 ± 7 μg/g pepper).

Conventional extraction methods for capsinoids suffer from several drawbacks which include low product yields and high solvent consumption. In addition, these extraction techniques are complicated and time-consuming and the low extraction yields lead to waste material which is a health hazard to personnel and deleterious to the environment.^51,52^ Such factors have led to intense study to design alternative extraction methods aimed at reducing solvent consumption, decreasing the time of extraction, and diminishing the production of waste material. Among these emerging environment-friendly technologies, microwave-assisted extraction (MAE) has proven to be an excellent approach to obtaining high yields of capsinoids from chili peppers.^45,51^ MAE has several advantages over conventional extraction techniques that includes shorter extraction times, lower solvent consumption, higher extraction efficiency, and a decrease in the quantity of undesirable waste products. The MAE technique involves applying energy via microwave radiation (frequency between 0.3 and 300 GHz) which interacts with the homogenized pepper extract to generate heat which accelerates the penetration of solvent into the homogenized pepper matrix, thereby increasing extraction efficiency. Optimization studies have shown that pure methanol is the best solvent for the MAE of capsinoids.^45^ HPLC analysis indicated the yield of **2** using MAE with methanol as the solvent was approximately 1404 ± 40 μg/g of pepper extract, which is about 1.5-fold higher than what is obtained with traditional extraction methods.

A second method for obtaining high yields of capsinoids from chili peppers is ultrasound-assisted extraction (UAE).^45,52^ This method is based on applying ultrasound waves with a frequency higher than 20 kHz at a specific frequency, amplitude, and wavelength to a fluid system consisting of crushed plant extracts and a suitable solvent to facilitate the extraction of capsinoids. The improved yield of capsinoids by UAE may be attributed to the hydrodynamic phenomena arising from “acoustic streaming” and “acoustic cavitation”. The application of ultrasound to the fluid system increases its flow velocity introducing turbulence to the fluid system,^52^ which generates alternate compression and rarefaction of the fluid that, in turn, results in vaporization of the solvent and the formation of gas bubbles. The gas bubbles subsequently implode, which increases the local temperature and pressure. The implosion of gas bubbles is termed “acoustic cavitation” and produces physical shearing forces which break the framework of the solid matrix of the macerated plant extract, facilitating increased penetration of solvent, enhanced extraction, and release of the compounds within the pulverized plant extract. Optimization of the UAE reaction conditions for the isolation of capsinoids from biquinho (*Capsicum chinense*) peppers involved lyophilizing the peppers and grinding them to a powder to increase the contact surface area between the solvent and the extract.^45^ After UAE, the solvent fractions were analyzed by HPLC methodology to quantify the concentration of the extracted **3**. A solvent mixture of 42% methanol and 58% ethanol produced the highest extraction yields for **2** (∼1324 ± 32 μg/g of pepper extract) by UAE methods. The efficacy of UAE is similar to MAE, and the yield of **2** using UAE was about 1.5-fold higher than conventional extraction procedures. A comparison of the efficacy of MAE with UAE to isolate capsinoids from biquinho peppers^45^ indicated that the UAE method was easier to implement, required a smaller initial investment in infrastructure and facilities, and was available to most laboratories. On the other hand, the amount of **2** obtained by MAE is slightly greater than that obtained by UAE. The process of UAE uses EtOAc as the solvent which cannot be directly introduced into the UV–visible reversed-phase chromatographic equipment. The EtOAc can potentially interact with and disturb the C18 stationary phase. It also has a high UV cutoff wavelength (256 nm), making detection on the UV–visible detector difficult.^53^ Therefore, UAE of **2** from biquinho peppers required an additional step where the EtOAc component was removed by evaporation.^45^ The process of MAE is more seamless and can be directly integrated into chromatographic techniques which reduces the total time required to extract the capsinoids.

Supercritical fluid extraction (SFE) is a new methodology that has been used to obtain high yields of capsinoids from chili peppers.^41,54^ Natural materials are usually found in three distinct phases: solid phase, liquid phase, and gaseous phase. A substance attains “supercritical state” if it is subjected to temperature and pressure beyond its critical point which is defined as the temperature (Tc) and pressure (Pc) above which discrete gas and liquid phases do not exist.^55^ A supercritical fluid displays dual properties of a gas (diffusion, viscosity, and surface tension) and a liquid (density and solubilization ability). Among all of the solvents capable of attaining “supercritical state”, CO_2_ has been used most frequently for the extraction of natural products. During the SFE process, the homogenized plant material is placed in a fixed bed and CO_2_ in the supercritical state is streamed through it.^56^ After completion of the extraction procedure, the CO_2_ is collected in a precipitator separator, where the solute is precipitated, accomplished by reducing the pressure of the chamber. The excess CO_2_ can be recycled and used for subsequent rounds of extraction. The nonpolar nature of CO_2_ makes it a suitable solvent for the extraction of capsinoids from chili peppers^56^ and this methodology was first used to extract capsinoids from biquinho peppers. Using this methodology, a substantial amount of **2** (27 mg/g of pepper extract) and **3** (1.85 mg/g pepper extract) was obtained when the temperature was held at 60 °C and the pressure was maintained at 15 MPa.^57^ The extraction efficiency of SFEs was almost double that of MAE and UAE. A comparison of these extraction methods is provided in Table 1.

**Table 1:** Comparison between MAE, UAE, and SFE with Conventional Extraction Procedures

method	strain of peppers used	raw material	solvent	purification method	yields of **2**, **3** and **4**	strengths	weaknesses	ref
traditional extraction	CH-19	freeze-dried peppers extracts	ethyl acetate	reverse-phase silica gel column chromatography	2 = 59 μg/g of pepper extract		low product yields	(40, 42−45, 49, 50)
	tabasco peppers	oleoresin		liquid–liquid partitioning	3 = 98 μg/g of pepper extract		high solvent use	
	germplasm 509–45–1 of the *Capsicum annum* pepper	chopped peppers		phenyl-hexyl column HPLC	2 ∼ 838 μg/g of pepper extract		large amount of waste material	
					3 ∼ 373 μg/g of pepper extract		risk to environment	
					4 ∼ 122 μg/g of pepper extract		risk to personnel	
							expensive	
MAE	biquinho peppers	freeze-dried peppers extracts	methanol, ethanol	reverse-phase UHP-HPLC	2 = 1404 μg/g of pepper extract	lower cost	none	(45)
						shorter extraction times		
						lower solvent use		
						higher extraction efficiency		
						decrease in waste products		
UAE	biquinho peppers	freeze-dried peppers extracts	methanol	reverse-phase UHP-HPLC	2 = 1324 μg/g of pepper extract	same as MAE	two-step process	(45)
SFE	biquinho peppers	freeze-dried peppers extracts	carbon dioxide	reverse-phase UHPLC	2 = 27 mg/g of pepper extract	solvent can be recycled	elaborate equipment needed	(41, 54, 57−60)
					3 = 1.85 mg/g of pepper extract	can be combined with UAE and HPE		
						higher extraction efficiency		
						capable for large-scale production of capsinoids		
						other strengths are same as MAE		

An advantage of SFE technology is that it can be combined with UAS to robustly increase the yields of bioactive phytochemicals isolated from peppers. A combination of SFE and UAS (called US-SFE) was applied to obtain capsinoids from Dedo de Moça peppers.^58,59^ The combination of the two techniques increased the extraction efficiency by 45%, and the yield of capsinoids was enhanced by 12%.^58^ Although, this process was not used to extract **2** and **3**, the data generated suggested that the yields of capsinoids may be increased by the application of US-SFE. An evaluation of the cost of using large-scale SFE along with high-pressure extraction (HPE) to obtain capsinoids from biquinho peppers^60^ found that the cost of manufacturing capsiate-rich extracts by the SFE + HPE technologies was considerably lower than the prevailing commercial prices for these extracts. In addition, SFE-based techniques are safer for both personnel and for the environment than conventional extraction methods.^60^ Such findings provide a strong incentive for adopting SFE-associated methodologies for commercial, large-scale manufacture of capsinoids from chili peppers.

### 3.2 Isolation of Resiniferatoxin from Plants

Resiniferatoxin (**5**, RTX) is a naturally occurring daphane diterpene compound in the fresh latex of the Euphorbia genus of cactus plants that was first isolated from the Moroccan cactus plant *Euphorbia resinifera*.^61^ It is an analog of **1** whose pain-relieving properties are mediated by the TRPV1 receptor,^62^ where it is approximately 1000-fold more potent than **1**.^63^ Several organizations are currently conducting clinical trials exploring the therapeutic potential of **5** as an analgesic to relieve the severe pain associated with cancers and arthritis.^62,64,65^ An acetone extract of *Euphorbia resinifera* and *Euphorbia unspina* isolated **5**; however, the extraction process was not described in adequate detail.^66^ The detailed procedure for isolating **5** used fresh latex of *E. resinifera* Berg as the starting material. The plant was pricked with a needle and the fresh latex collected on preweighed filter papers which were stored at 4 °C until extraction. The extracts were filtered through TLC-grade silica gel to remove the gummy material in the extract. The clear filtrate obtained was dried to yield a yellow-colored amorphous semisolid material that was dissolved in CH_3_CN and recrystallized to obtain white-colored crystals. The mother liquor left after crystallization was purified to obtain **5**.^67^ However, the yield of pure **5** obtained by this process was very low, about 0.002%, for two predominant reasons. The first was the low abundance of **5** in *E. resinifera* Berg. The second was the fact that conventional column chromatography to obtain pure **5** resulted in fractions where **5** was contaminated with ingenol and 12-deoxyphorbol esters. A complex HPLC purification protocol was used to obtain pure **5** which further lowered the yields of the pure compound.

**Figure 5:**
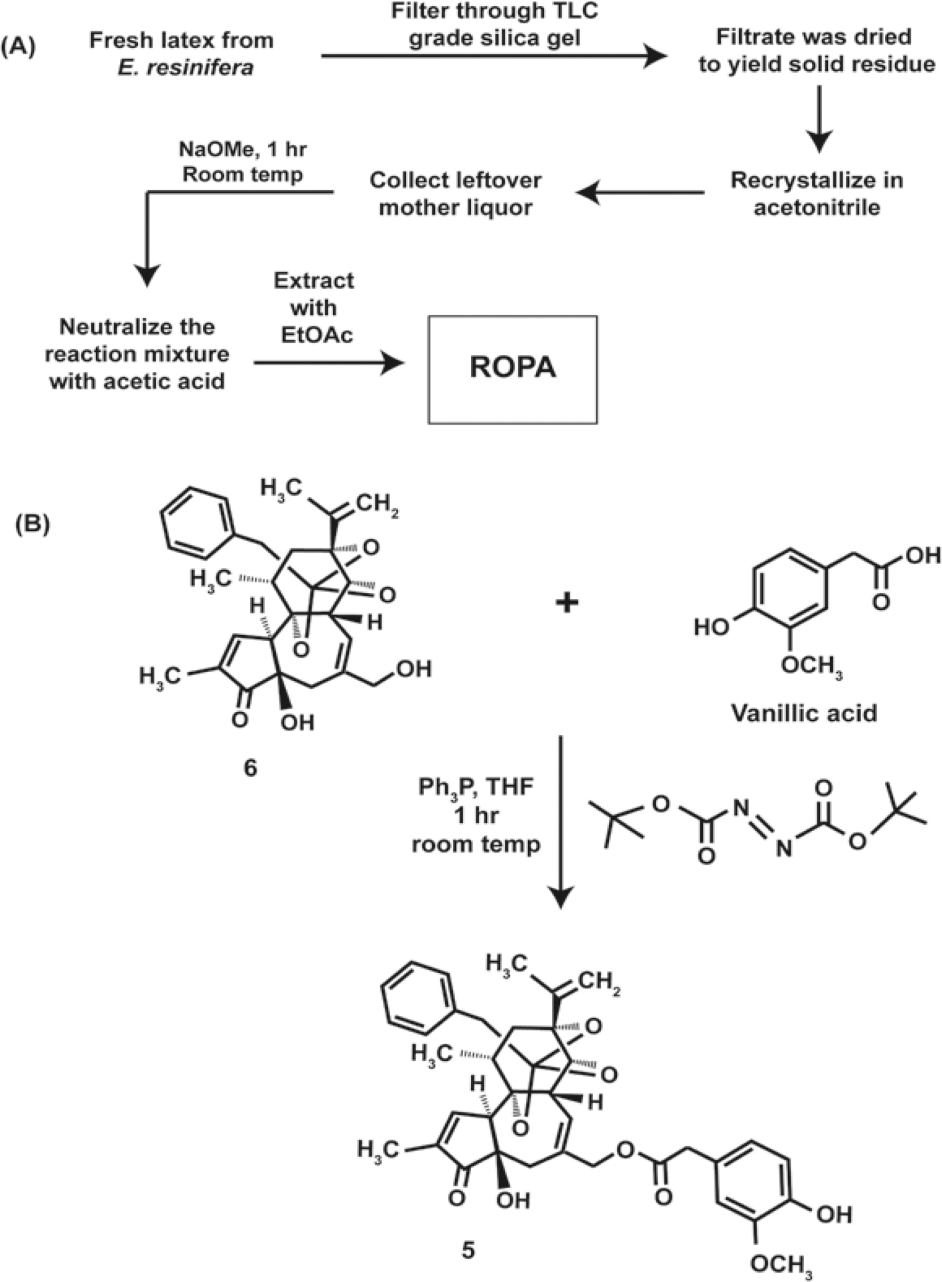
(A) Large-scale isolation of **5** from *Euphorbia resinifera*. The crude extract obtained from the plants was hydrolyzed to give ROPA. (B) ROPA was converted back to resiniferatoxin by chemical synthesis.

Subsequent attempts to scale up this extraction process to obtain a greater amount of **5** were thwarted by the high irritant properties of the diterpene which made it very difficult to scale up conventional chromatographic procedures.^67^ An alternate route was explored whereby the **5** in the mother liquor from the CH_3_CN extraction was hydrolyzed to generate **6** (Figure 5A). Compound **6** is devoid of TRPV1-binding activity and has very weak pungency properties and was readily purified by column chromatography and converted back to **5** using either a nucleophilic displacement reaction or a Mitsunobu esterification procedure (Figure 5B).

## 4 Chemical Synthesis Methods for Natural Region B Capsaicin Analogs

### 4.1 Chemical Synthesis of **2** and **3**

**Scheme 1:**
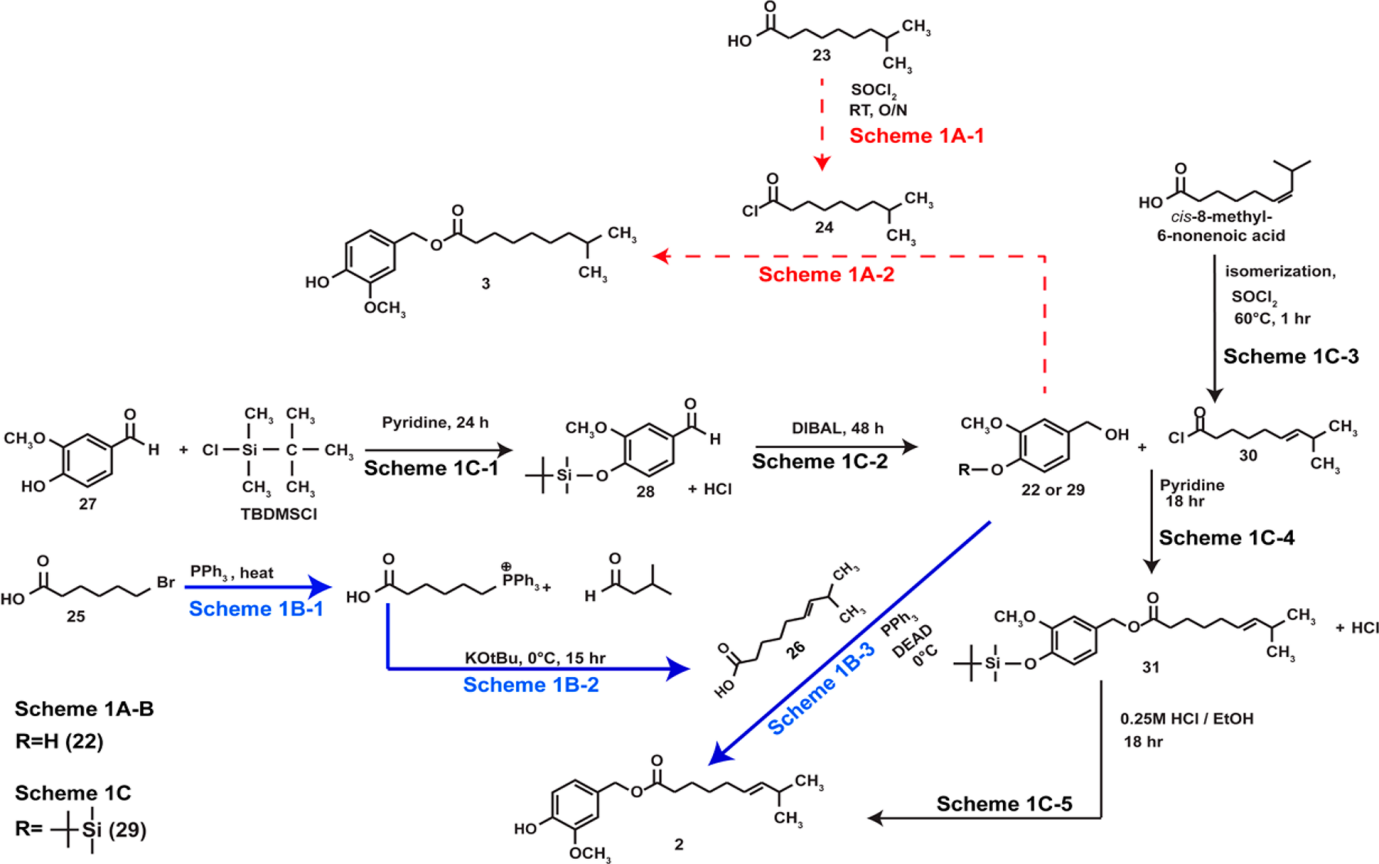
Approaches to the Chemical Synthesis of **2** and **3**

The reaction between vanillyl alcohol (**22**) and a fatty acid chloride (or a fatty acid in the presence of an activating moiety) is the most prevalent chemical synthesis strategy to obtain region B capsaicin analogs. The capsinoid **3** was obtained by a acylation reaction between **22** and 8-methylnonenyl chloride (**24**), as depicted in Scheme 1A (red dotted arrows).

**Scheme 2:**
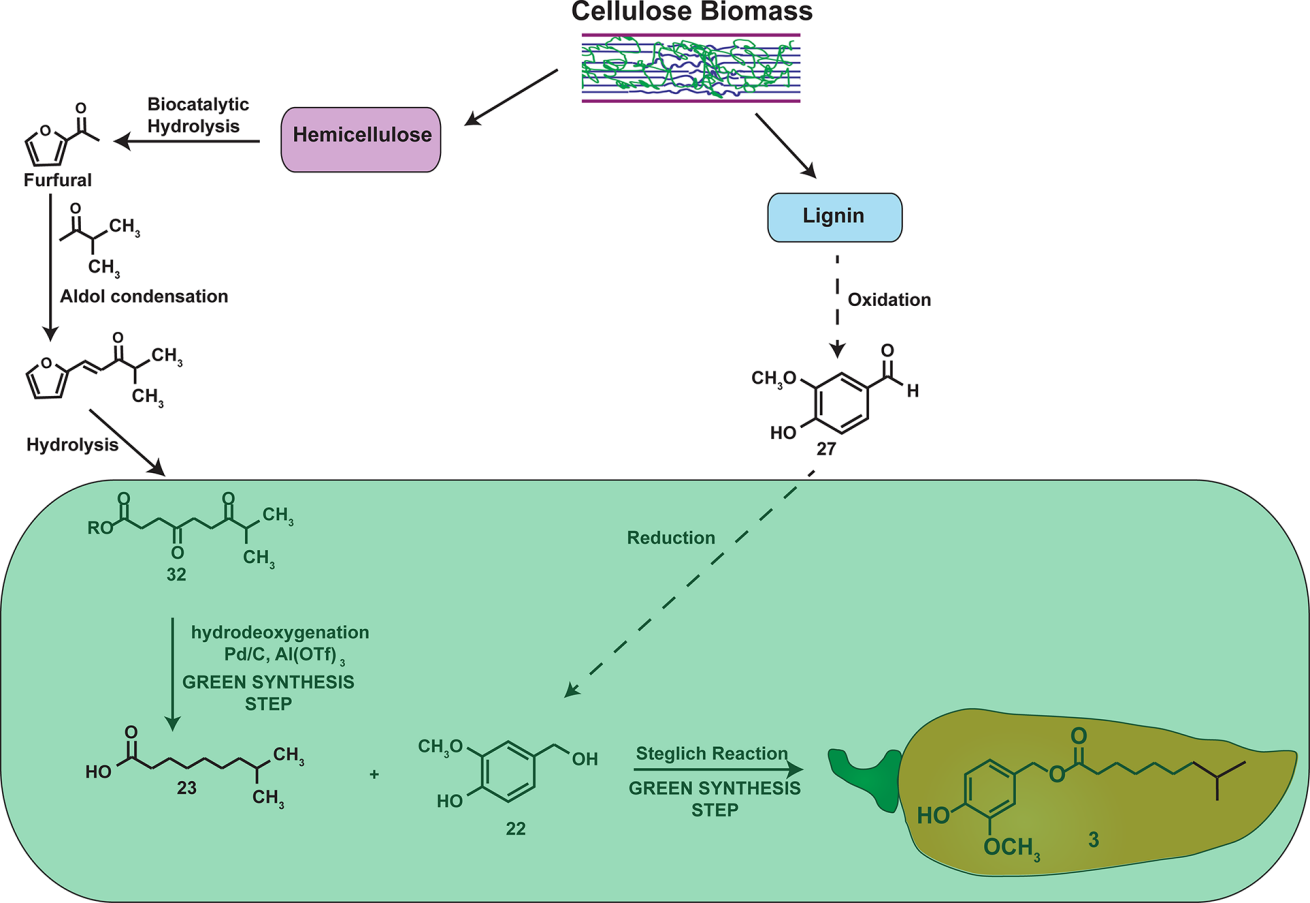
Green Synthesis of **3** from Cellulose Biomass

**Scheme 3:**
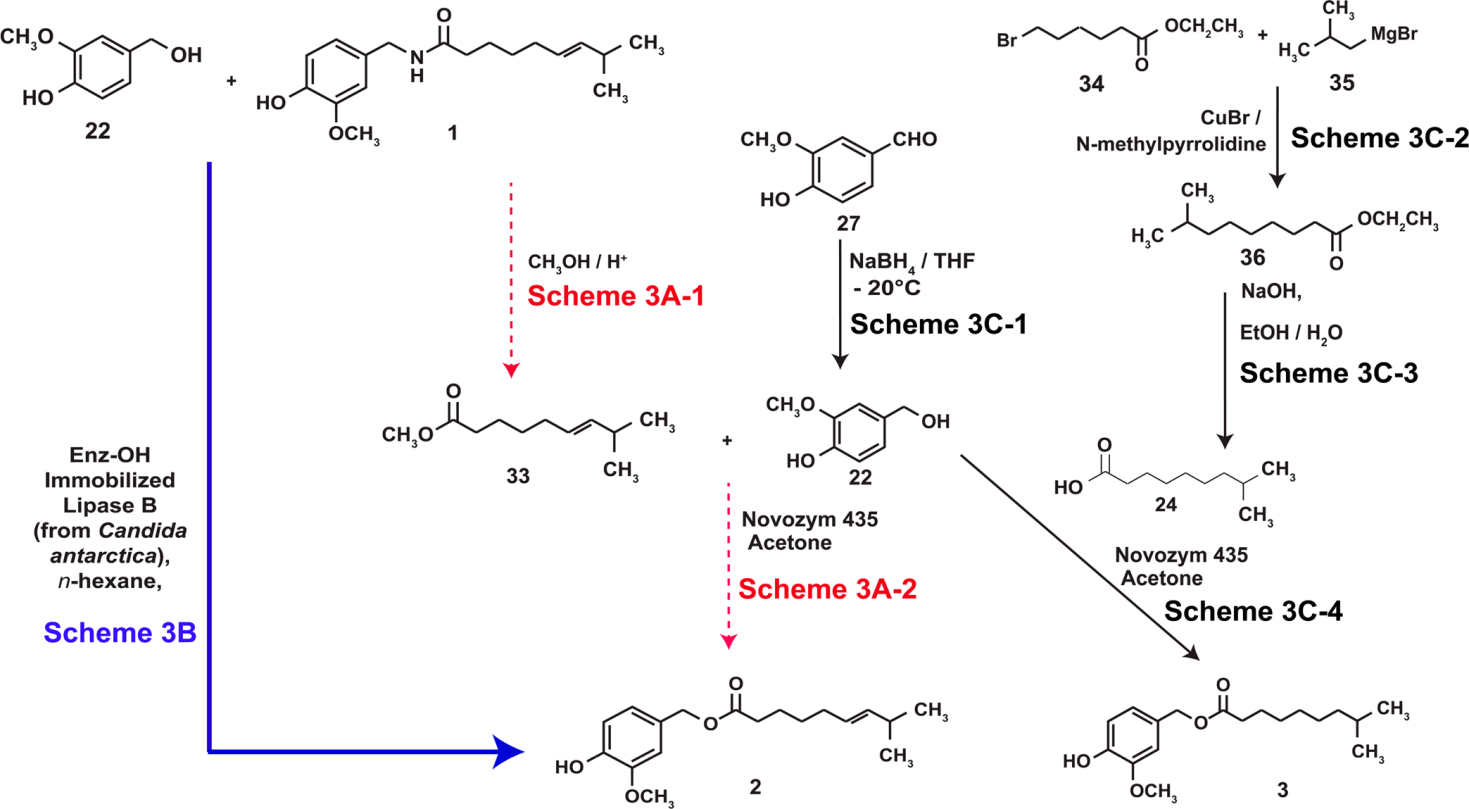
Enzymatic Synthesis of **2** and **3**

The 8-methylnonanoyl chloride (**23**) was generated from 8-methylnonenoic acid (**23**) by overnight exposure to SOCl_2_ (Scheme 1A-1), and the oily product reacted with the aromatic alcohol **22** to obtain **3** (Scheme 1A-2).^40^ Similarly, the first step for the synthesis of **2** was to prepare the 8-methyl-6-nonenoic acid (**26**), as depicted in Scheme 1B (blue arrows).^68^ The carboxylic acid **26** was obtained from 6-bromohexanoic acid (**25**) by a sequence involving a Wittig reaction between the triphenylphosphonium salt derived from **25** (Scheme 1B-1) and isobutyraldehyde (Scheme 1B-2, blue text, blue arrows). Finally, **26** was reacted with alcohol **22** to yield **2** (Scheme 1B-3). An analogous chemical synthesis strategy was used to synthesize **4**.^69^

**Scheme 4:**
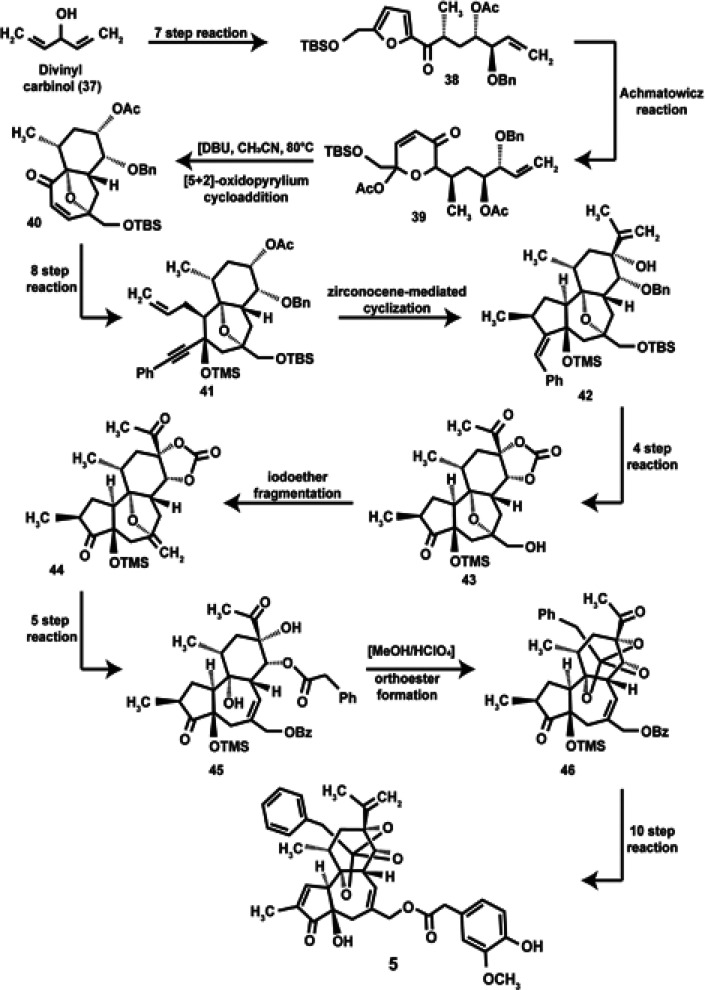
Chemical Synthesis of **5**

**Scheme 5:**
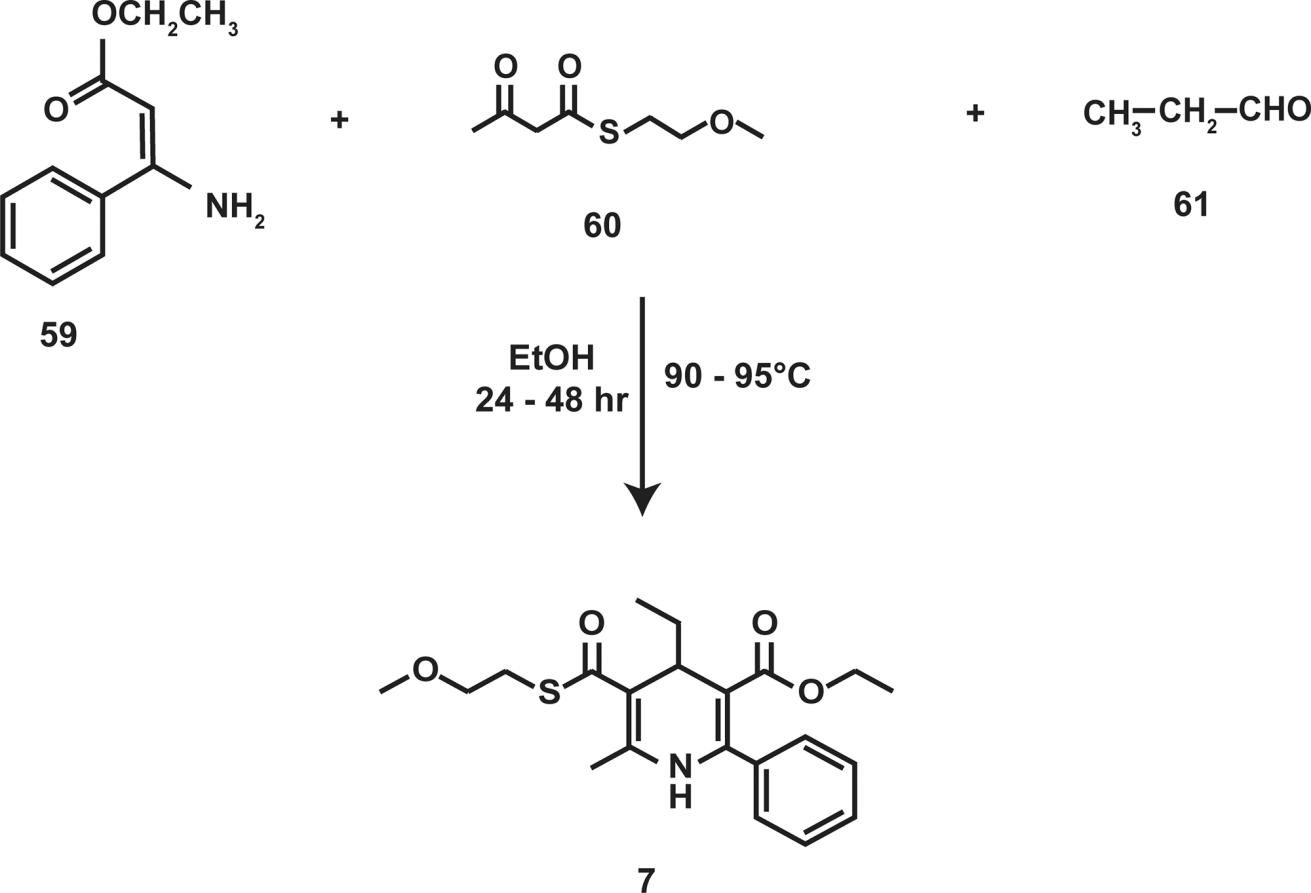
Chemical Synthesis of **7**

A caveat of both these synthetic methodologies is that the phenolic–OH of vanillyl alcohol can react with the fatty acyl chloride or activated fatty acid derivative to generate a substantial amount of byproduct. Silylation of the phenolic–OH group is one strategy to avoid these byproducts, and a high-yielding, multistep synthesis of **2** was achieved using *t*-butyldimethylsilyl chloride (TBDMSCl) to protect the phenolic −OH group of vanillin (**27**, Scheme 1C).^70^ A strength of this methodology is that the reagents used in the synthesis are inexpensive. The initial step of this synthesis protocol involved the reaction between **27** and TBDMSCl (Scheme 1C-1) to generate 4-*t*-butyldimethylsilyloxy-3-methoxybenzaldehyde (**28**).^70^ The carbonyl group of **28** was reduced to the corresponding alcohol using di-isobutylaluminum hydride (DIBAL) to obtain pure 4-*t*-butyldimethylsilyloxy-3-methoxybenzyl alcohol (**29**), as summarized in Scheme 1C-2. Finally, *trans*-8-methyl-6-nonenoyl chloride (**30**), obtained from the commercially available *cis*-8-methyl-6-nonenoic acid by the method depicted in Scheme 1C-3, was reacted with **29** to obtain 4-*t*-butyldimethylsilyloxy-3-methoxybenzyl-*E*-8-methyl-6-nonoate (**31**, Scheme 1C-4).^70^ Desilyation of **31** using 0.25 M HCl/EtOH) yielded pure **2** (Scheme 1C-5). Additional advantages of this multistep synthesis protocol were (1) the reactions were easy to perform and gave excellent yields, (2) the reaction products were easily purified, and no significant secondary byproducts were observed, and (3) all of the reactions were regioselective. Finally, this chemical synthesis route can be adapted to create **3** and **4**.

Although effective, the traditional chemical synthetic methods to generate capsinoids also suffer from some disadvantages. Installation of protecting groups are needed prior to the esterification reaction to improve yields and prevent formation of unwanted byproducts. Moreover, the catalysts and condensing reagents used in these reactions need to be handled with care because they are often corrosive. Such drawbacks can be circumvented by the use of “green chemical synthesis methods” which are safer for humans and less hazardous for the environment. An innovative “green synthetic method” to obtain **3** used cellulose biomass as the starting material. Cellulose biomass is composed of two kinds of carbohydrate polymers, cellulose and hemicellulose, and an aromatic-rich polymer named lignin. The authors generated **23** from hemicellulose^71^ by hydrolytic degradation to furfural using an *E. coli*-based biosynthesis approach, as summarized in Scheme 2. The furfural was subjected to an aldol condensation reaction with 3-methylbutan-2-one followed by hydrolysis of the enone to obtain the diketo ester **32** which was reduced to obtain the acid **23**.^71^ The phenolic long chain polymer lignin is a renewable source of aromatic compounds like **27**. Traditionally, the isolation of **27** from lignin is an inefficient process and generates very low yields of **27**. The catalytic oxidation of lignin using nitrobenzene or copper as catalyst produces excellent yields of **27** (Scheme 2). The reduction of **27** generated **22** (Scheme 2), which was coupled with **23** under Steglich reaction conditions to obtain **3** in high yields. Another advantage of this green synthesis methodology is that it uses inexpensive and sustainable materials like cellulose biomass.

## 5 Enzymatic Synthesis Methods for Region B Capsaicin Analogs

Enzyme-catalyzed biotransformation reactions are a highly promising strategy for the large-scale synthesis of chemical compounds.^72,73^ In particular, the enzymes of the lipase family have been recognized as efficient catalysts for the synthesis of pharmacologically active esters.^72,74^ Such enzymatic reactions are usually performed in anhydrous organic solvents, and the immobilization of enzymes on polymers has facilitated the large scale production of biologically active esters. The biocatalysts Lipozyme IM20 and Novozym 435 are prepared by immobilizing *Candida antarctica* lipase B on macroporous ion-exchange resins and polyacrylic polymers, respectively.^75^ Of these two, Novozym 435 exhibits better catalytic activity over a wide range of organic solvents and reaction temperatures.

The major strengths of lipase-catalyzed synthesis of capsinoids include the mild reaction conditions, a high rate of conversion of substrates to the desired product, the absolute regioselectivity of the reactions, the absence of any byproducts and high isolated yields of the final products.^72,74^ In addition, just like the “green synthesis protocols”, the use of enzymatic synthesis reactions to access capsinoids is less hazardous to personnel, environmentally friendly, is associated with low production costs, and purification of the product is straightforward. The first attempt to synthesize **2** enzymatically was via the two-step reaction summarized in Scheme 3. The acyl donor for **2**, methyl (6*E*)-8-methyl-6-nonenoate (**33**), was obtained by methanolysis of capsaicin (Scheme 3A-1).^76^ Condensation of alcohol **22** with ester **33** in the presence of Novozym 435 in dioxane as solvent provided **2** in 80% isolated yield, as summarized in Scheme 3A-2. No undesirable side products were observed in this reaction and high amounts of **2** could also be obtained when using dihydrocapsaicin as the starting material in the reaction.

A drawback to the synthesis methodology outlined in Scheme 3A is that it is a multistep process, an observation that underscores the need for a single-step enzyme-catalyzed synthesis of capsiate. A direct one-step synthesis of **2** was achieved through a lipase-catalyzed alcoholysis-type dynamic transacylation reaction, starting with equimolar concentrations of capsaicin (**1**) and vanillyl alcohol (**22**) as the acyl-acceptor (Scheme 3B).^77^ The biocatalyst for this reaction was immobilized lipase from *Candida antartica*. A noteworthy observation was that the formation of the acyl-enzyme complex and the subsequent alcoholysis reaction is dependent on the nature of the reaction media. The use of a polar solvent, namely 2-methyl-2 butanol, did not yield any reaction products.^78^ On the other hand, the dynamic transacylation reaction proceeded efficiently in a nonpolar solvent like *n-*hexane. Such observations may be explained by the fact that the chemosensitivity of the lipase may be dependent on the thermodynamic properties of nonpolar solvents (like *n*-hexane), which allow direct conversion from an amide to an ester without any intermediates.

A large-scale synthesis methodology of **3** is based upon a Novozym 435-catalyzed esterification reaction of **22** with **24** (Scheme 3C–4).^79^ Although **22** is available commercially, it can be readily prepared by the reduction of **27** using NaBH_4_ as the reducing agent (Scheme 3C-1). Subsequently, the large-scale synthesis of **24** was accomplished by a cross-coupling reaction between the ethyl ester of 6-bromohexanoate (**34**) and iso-butylMgBr (**35**) using CuBr as a catalyst to afford ester **36** (Scheme 3C-2). Saponification of the reaction product yielded **22** (Scheme 3C-3).^79^ Finally, the esterification reaction between alcohol **22** and acid **24** was catalyzed by the immobilized lipase (Novozym 435) to obtain pure **3** (Scheme 3C-4). This chemo-enzymatic synthesis procedure could be modified to produce excellent yields of **2** and **4**.^79^

Among the capsinoids, **4** displays the maximal anticancer activity in cell culture and mouse models of leukemia and skin cancers (refer to section 9.1.1).^69,80^ Despite such promising data, there are only a few studies that have investigated efficient methods for the large scale production of **4**. The yield of **4** from traditional extraction methods is poor; there are no reports involving the use of newer technologies like UAE, MAE, or SFE for the extraction of **4**, and there are only a small number of descriptions of the chemical or enzymatic synthesis of **4**. It is hoped that future studies will discover novel methods to efficiently isolate or synthesize **4** in high yield and purity.

## 6 Chemical Synthesis of RTX (**5**)

The complex structure of **5** represents a formidable challenge in organic chemical synthesis. In particular, the generation of the densely oxygenated *trans*-fused 5/7/6-tribocycle (ABC-ring) is the most challenging chemistry (Table 2). The first total synthesis of **5** comprised of 45 steps and used 1,4-pentadien-3-ol (divinyl carbinol, **37**) as the starting material (Scheme 4).^81^

**Table 2:** Comparison between Chemical Synthesis Procedures to Obtain **5**

method	starting reactants	number of steps	highlights of the synthesis	ref
Wender et al. (1997)	37	45	[5 + 2}-oxidopyrylium cycloaddition	(81)
			zircocene-mediated cyclilization	
			reductive iodoether fragmentation	
Hashimoto et al. (2017)	47, 48, 49	41	two free radical condensation reactions	(143)
			[3,3] sigmatropic rearrangement	
			free radical-catalyzed 7-endo cyclization	
			VAZO initiators for free radical coupling reactions	
Hikone et al. (2022)	51, 52, 53	27	radical allylation	(144)
			stille coupling	
			photocatalyzed decarboxylative 7-endo radical cyclization	
			yield of RTX was 40-fold higher than Wender et al. (1997)^81^ and Hashimoto et al. (2017)^143^	
Vasilev et al. (2022)	55, 56,57, 58	15	rapid assembly of 5,7-hydrazulene core	(145)
			stereoselective aldol condensation of two chiral fragments	
			Kagan’s reagent used to achieve radical cyclization	
			formation of caged orthoeaster via a unique epoxide ionization cascade	

Subsequently, the total synthesis of **5** has been accomplished by four distinct chemical synthesis routes (Schemes 1–3 of the Supporting Information). These four synthesis methods are compared in Table 2.

## 7 Chemical Synthesis Methods for Synthetic Region B Capsaicin Analogs

### 7.1 Allosteric TRPV1 Ligand MRS1477

**Figure 6:**
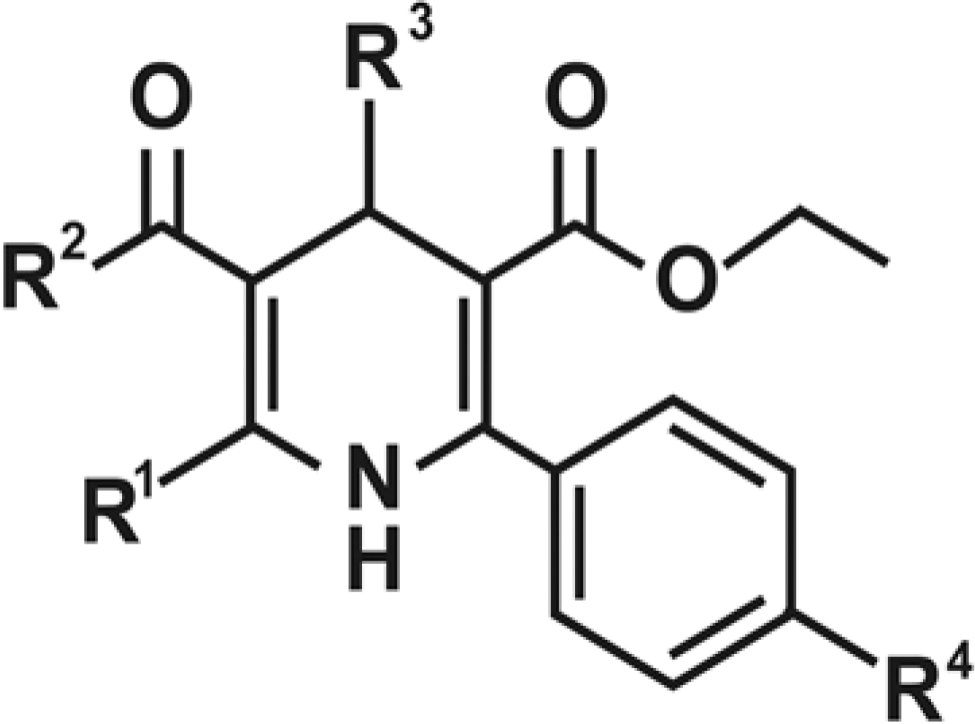
General structure of 1,4-DHP capsaicin analogs.

The TRPV1 receptor plays a vital role in the regulation of acute/chronic inflammatory pain. The serendipitous observation that the dihydropyridine (DHP)-based calcium channel antagonist nifedipine could regulate the biological function of the activated TRPV1 receptor suggested that 1,4-DHP-based derivatives may be a promising scaffold for the design of allosteric TRPV1 ligands.^82^ Substituted 1,4-DHPs have been found to enhance TRPV1 activity in several experimental models. However, these compounds target the activated TRPV1 receptor^82^ but exhibit minimal/no intrinsic agonist activity of their own. This novel class of TRPV1 enhancers acts via an allosteric mechanism to prevent the opening of the pore domain of the receptor.^82^ The general structure of these 1,4 DHP allosteric enhancers in presented in Figure 6.

The 1,4-DHP-based allosteric capsaicin analogs were tested for their ability to enhance capsaicin-induced ^45^Ca^2+^ uptake in TRPV1-NIH3T3 mouse fibroblasts and cultured dorsal root ganglion neurons that express TRPV1.^82^ SAR studies showed that the presence of a thioester at position R_2_ produced better TRPV1 enhancers than an ester group. Similarly, DHP derivatives containing a phenyl group at R_4_ and small alkyl groups at R^2^ and R^3^ enhanced capsaicin-induced calcium flux at the TRPV1 receptor. MRS1477 (**7**) was identified as the most potent allosteric TRPV1 agonist^82^ that stimulated capsaicin-induced ^45^Ca^2+^ uptake in TRPV1-NIH3T3 cells by greater than 6-fold relative to the controls. The synthesis of **7** involved the condensation of a β-enaminoester (**59**), a β-ketoester (**60**), and propionaldehyde (**61**), as shown in Scheme 5.

### 7.2 Capsaicin Analogs Containing the 1,3-Benzodioxole Bicyclic Motif

**Scheme 6:**
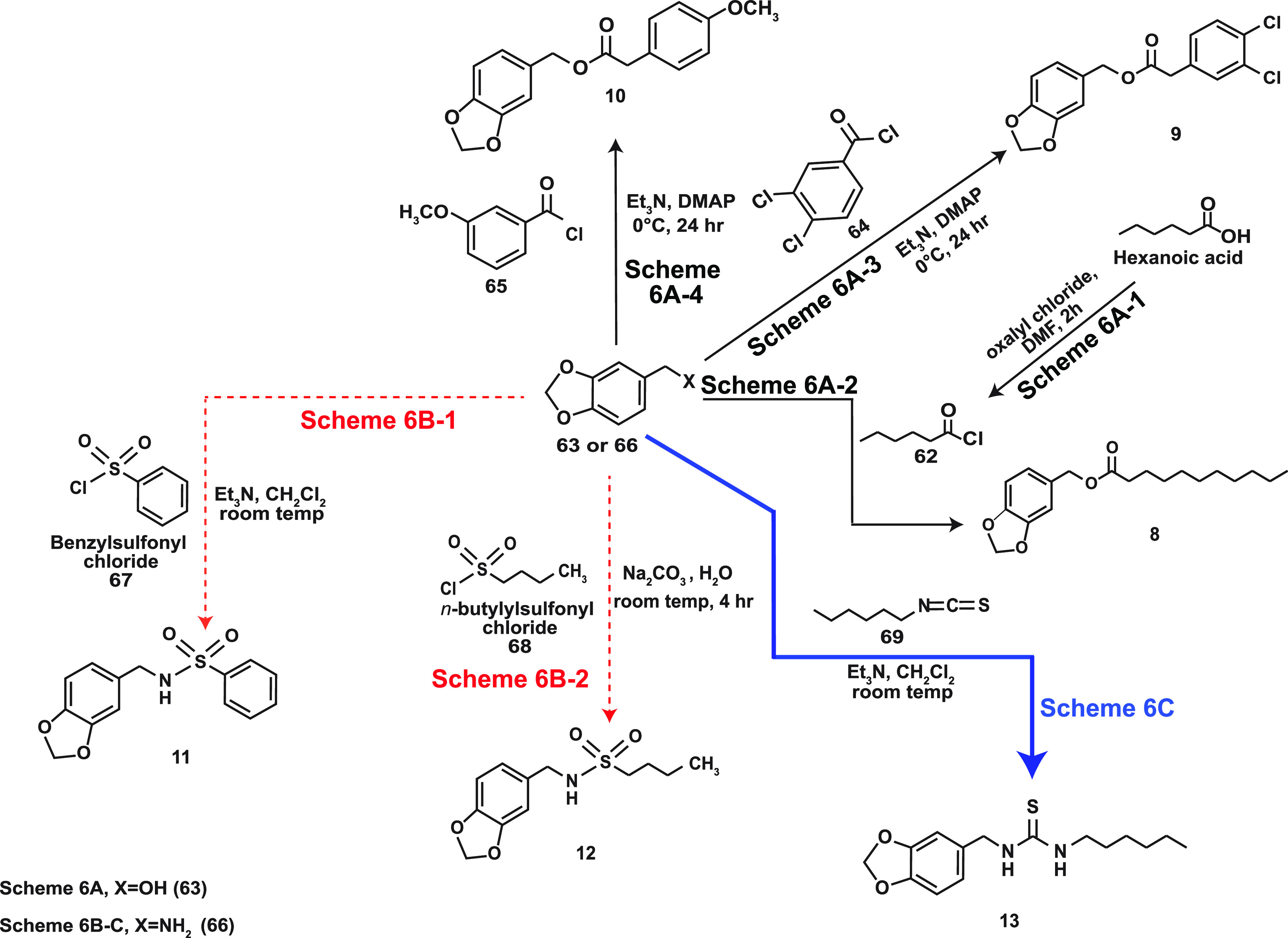
Chemical Synthesis of 1,3-Benzodioxole Motif Containing Region B Capsaicin Analogs

Computational modeling using an *in silico* ligand-based drug design strategy represents an attractive approach for creating novel capsaicin analogs.^83^ The BMH series of region B capsaicin analogs, namely BMH (**8**), BMDPh (**9**), and BMDPh-O (**10**), were designed using *in silico* molecular modeling approaches. The amide group in region B of capsaicin was replaced by an isosteric ester group. Bioisosterism is a useful strategy to improve the potency, selectivity, and pharmacokinetics of key compounds.^800^ In a large number of compounds, the replacement of an amide bond by suitable bioisosteric moieties like an ester, sulfonamide, or thiourea maintain similar steric and hydrogen-bonding properties of the prototype molecule. The lipophilic chain in region C was replaced by a range of alkyl and aryl groups designed to maintain the hydrophobic character of this specific region of the pharmacophore. The synthesis of 1,3-benzodioxole motif-containing region B capsaicin analogs is summarized in Scheme 6. Hexanoic acid was treated with oxalyl chloride in the presence of DMF to obtain the corresponding acid chloride **62** (Scheme 6A-1).^83^ Subsequently, benzo[*d*][1,3]dioxol-5-yl-methanol (**63**) was reacted with **62** in the presence of DMAP as a catalyst to obtain **8** (Scheme 6A-2). The capsaicin analogs **9** and **10** were obtained from the reaction of **63** with 3,4-dichlorobenzoyl chloride (**64**) and 3-methoxybenzoyl chloride (**65**), respectively (Scheme 6A-3, A-4).

An important class of region B capsaicin analogs are the constrained sulfonamides represented by RPF 101 (**11**) and RPF 151 (**12**).^84,85^ The sulfonamide moiety is stable toward chemical hydrolysis and physiological enzyme-mediated degradation processes.^86,87^ The capsaicin analog **11** was created by a direct reaction between piperonylamine (**66**) and benzylsulfonyl chloride (**67**), as depicted in Scheme 6B-1. Molecular modeling studies suggested that the presence of the phenyl group in region C of **11** maintained the homology and rigidity of the molecule while endowing a lipophilic character. However, a caveat with **11** was that it was insoluble in common aqueous and organic solvents.^84^ This led to the development of **12** in which the C-terminal phenyl group was replaced by an *n*-butyl group which improved its aqueous solubility properties^85^ (Scheme 6B-2). The constrained capsaicin analog **13** contained a bioisosteric thiourea group in region B. Region C of **13** contained a hexyl moiety which bequeathed the compound with hydrophobic properties.^88^ The synthesis of **13** involved a direct reaction between **66** and *n*-hexyl isothiocyanate (**69**), as summarized in Scheme 6C.

A common feature of all of these compounds was that they contain the 1,3-benzodioxole bicyclic motif in region A of their structure. The 1,3-benzodioxole bicyclic system is a privileged structural feature present in a number of natural anticancer agents, including the podophyllotoxin derivatives etoposide and tenoposide.^89,90^ However, a problem with molecules that contain the 1,3-benzodioxole bicyclic motif is that they can be metabolized by cytochrome P450 enzymes into tight-binding inhibitors. Cytochrome P450-catalyzed metabolism of the 1,3-benzodioxole substituent can result in the generation of a carbene intermediate that binds tightly to the Fe atom of the enzyme.^91,92^ After degradation of the carbene, a catechol metabolite is released that can be subject to CYP 450-mediated oxidation to produce an orthoquinone species, which is chemically reactive and has been associated with toxicity. Thus, the 1,3 benzodioxole motif is regarded as a structural alert that has the potential to be a source of toxicity and drug–drug interactions.^91,92^

### 7.3 Capsazepine and its Analogs

Capsazepine (**17**) is a synthetic thioureidic analogue of capsaicin that was discovered and characterized by Sandoz^93^ and is a potent TRPV1 antagonist. In this molecule, region A of the pharmacophore of capsaicin is constrained by the introduction of a five-membered, 5,6-isoindoline or a six-membered tetrahydroisoquinoline ring motif (Figure 1). It was observed that the presence of the catechol function in the 6,7-position retained the biological activity of the progenitor compound. The presence of the 4-chlorophenethyl thiourea group resulted in increased affinity of **17** for the TRPV1 receptor. A remarkable discovery was the fact that replacement of the five-membered isoindoline structure (in region A) by the six-membered tetrahydroisoquinoline ring (which entailed the addition of a single methylene group into the ring structure) transformed the compound from a TRPV1 agonist to a TRPV1 antagonist.^93^

**Figure 7:**
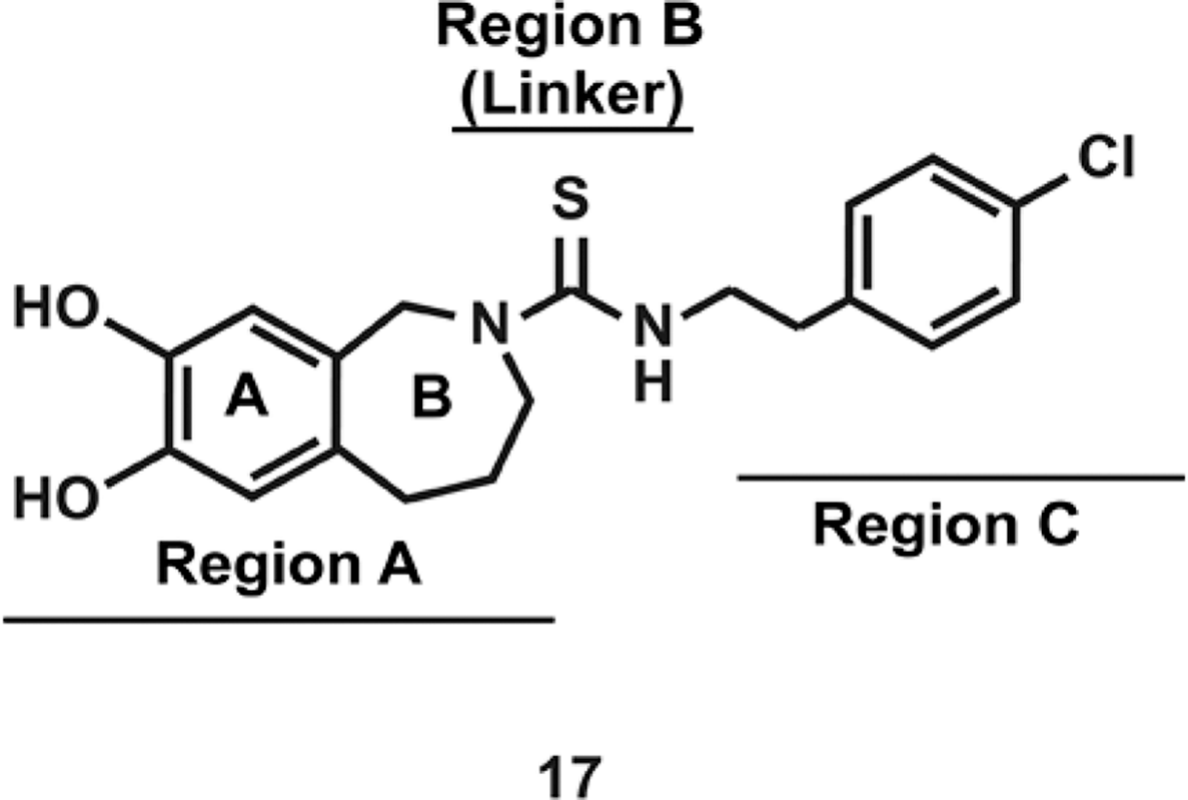
Pharmacophore of **17**.

The pharmacophore of **17** is comprised of four regions, as summarized in Figure 7. Region A contains the catechol moiety of the A-ring as well as the 2,3,4,5-tetrahydro-1*H*-2-azepine moiety (the B-ring) of capsazepine (**17**). Region B is the thiourea linker group while the C-terminus 4-chlorophenethyl group forms region C of the molecule.^93,94^ The conformational constraint conferred by the fused ring motif is important for the pharmacological activity of **17**. Thus, capsazepine analogs containing a six-membered 1,2,3,4-tetrahydroisoquinoline B-ring display better growth-suppressive activity than the corresponding 5,6 isoindoline, five-membered ring derivatives. This observation is exemplified by the capsazepine analog **19** (Figure 3), which displayed robust growth suppressive activity in HeLa human cervical cancer cells; the IC_50_ (obtained by the MTS viability assay) was lower than 10 μM. However, when the six-membered 1,2,3,4-tetrahydroisoquinoline B-ring of **19** was replaced by the five-membered isoindoline group, the IC_50_ was observed to be greater than 100 μM in HeLa cells. Systematic SAR studies led to the design of the constrained capsazepine analogs **18**–**21** (Figure 3).^95,96^

The attachment of a phenyl group to the tetrahydroisoquinoline ring increased the growth-suppressive activity of the resulting capsazepine analog **21**, whereas the corresponding isopropyl derivative was inactive. MTS cell viability assays revealed that the IC_50_ of **21** was lower than 5 μM in HeLa human cervical carcinoma cells.^95^ In contrast, when an isopropyl group was attached to the tetrahydroisoquinoline the IC_50_ values were greater than 50 μM in HeLa cells. The region B of these capsazepine analogs was identical to the parent compound. In region C, shortening the phenethyl chain to benzyl- or *para*-chlorobenzyl produced capsazepine analogs with robust growth-inhibitory activity. A total of 37 capsazepine analogs were created and their physicochemical properties, namely, log *P* and tPSA (total polar surface area) were determined.^95^ MTS assays demonstrated that the compounds **18**–**21** had the lowest IC_50_ values in HeLa human cervical carcinoma cells. The compounds **18**–**21** had log *P* values which closely resembled **17** and were below 5 (which is an indicator of a good drug candidate). Similarly, the tPSA value of **18**–**21** were similar to **17**. These tPSA values (of **18**–**21**) were below 100, which suggested these molecules would have good cell-membrane permeability properties.^95^ On the basis of the data obtained, four compounds, **18**–**21**, were selected for downstream biological testing to assess their potential as anticancer drugs.

## 8 Stability and Metabolism of Capsaicin Region B Analogs

The gastrointestinal metabolism of **2** was investigated in Sprague-Dawley rat models. Compound **2** was administered at a dose of 20 mg/kg bodyweight as a suspension in olive oil using a gastric feeding tube (for a period of 3 days), and the urine and feces were collected using a metabolic chamber every 24 h for 2 days.^97^ The article does not shed light on the reasons for using 3 days as the experimental time point for the pharmacokinetics experiments. A possible explanation of this study design may lie in a previously published article by the same research group where the oral administration of the related compound **1** was studied in Sprague-Dawley rats for a period of 3 days.^98^ It is likely that the metabolism of **2** was anticipated be somewhat similar to **1**, so the same study design was used to examine the metabolism of **2** in Sprague-Dawley rats. HPLC analysis revealed the absence of intact **2** in the urine or feces within 48 h. Compound **2** was rapidly metabolized *in vivo* to yield **22**, vanillic acid (**70**), and glucuronide conjugates of **22** and **70** in the urine. The glucuronidated conjugates of **22** and **70** accounted for 80% of the metabolites of **2** that were excreted in the urine.^97,99^ In the feces, there was no trace of intact **2**, **22**, or **70**. The gastrointestinal absorption of **2** in Sprague-Dawley rats was also investigated following a dose of 3 mg of **2** admixed in the diet and orally administered to rats following an overnight fast. After 1 h, almost the entire amount of **2** was found in the stomach and no trace of **2** was found in the duodenum, jejunum, ileum, cecum, or in the large intestine. No trace of **2** was found in the portal vein.^97,99^ After 3 h, the content of **2** in the stomach decreased and a small amount of **2** was discovered in the large intestine. A meager quantity of metabolites of **2** were identified in the alimentary canal. These observations suggest that the bioavailability of capsaicinoids *in vivo* is quite low. Such data has led to intense research focused on the design and synthesis of long-acting capsaicin analogs (with increased bioavailability and half-life) and the development of sustained release formulations of these compounds.

The pharmacokinetic profile of **3** following oral administration to seven-week-old Sprague-Dawley rats was determined by administering a ^14^C-labeled compound^100^ mixed with 5 mL of midchain triglycerides via a gastric feeding tube. The dose of **3** used in these studies was a 10 mg/kg body weight which was equivalent to 11.6 MBq of 3/5 mL bodyweight. The tissue distribution of radiolabeled **3** was monitored from 15 min to 24 h postdose.^100^ No intact **3** was observed in the plasma between 15 min and 6 h postdosing. However, metabolites of **3**, namely **22**, **70**, glucuronidated vanillyl alcohol and vanillic acid glucuronide, and sulfated vanillyl alcohol and sulfated vanillic acid were present in the plasma at measurable quantities. The highest concentrations of radioactive metabolites were found in the skin followed by the blood and kidneys at 2 h postdose.^100^ Almost the entire radioactivity was excreted in the urine and feces after 72 h.

**Figure 8:**
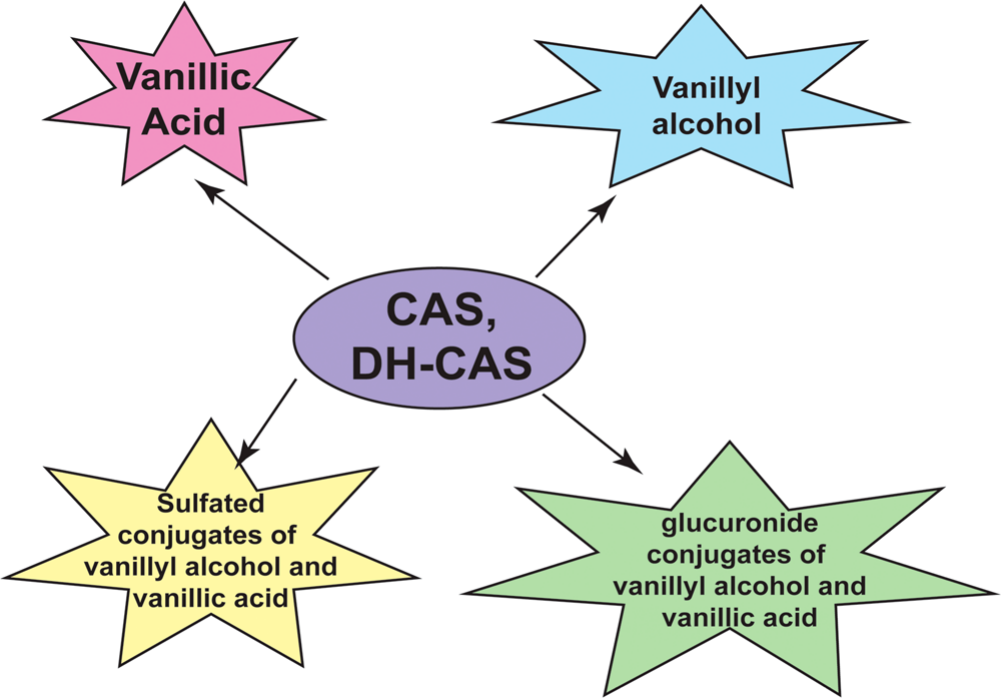
Metabolism of **2** and **3***in vivo*.

The metabolism of capsinoids is summarized in Figure 8 which shows that the major circulating metabolites of capsinoids are free and conjugated **22** and **70**. The bioavailability of CH-19 pepper extract, which contains a mixture of capsinoids, was examined by administering a single dose of the sweet pepper extract in midchain conjugated forms of **22** and vanillic acid triglycerides (containing 10–100 mg of capsinoids/kg body weight) to seven-week-old Sprague-Dawley rats via oral gavage.^101^ No trace of intact capsinoids were found in the portal and systemic blood between 5 min and 4 h postdose; rather, robust amounts of free and sulfate-conjugated vanillyl alcohol was present in the blood. On the basis of the above-mentioned studies, it appears that orally administered capsinoids are metabolized to **22** in the gastrointestinal tract and in the alimentary canal. The oral mucosa contained only conjugated vanillyl alcohol. Both the free and conjugated forms of **22** are transported to the liver via the portal vein where the **22** was biotransformed into glucuronidated and sulfated derivatives. Sulfated vanillic acid was also detected in the liver. Compounds **70** and **22** display potent growth-suppressive activity in human breast cancer, colon cancer, prostate cancer and melanoma, combat inflammation, block tumor angiogenesis, and the migration of human cancer cells. In addition, scientists have postulated that the combination of oxaliplatin and **70**, coformulated in sustained release polymeric micelles, may have potential applications in the treatment of colon cancer. These observations raise the possibility that capsinoids may function as pro-drugs **22** or **70**.^99,101^

## 9 Anti-Neoplastic Activity of Capsaicin Region B Analogs

**Figure 9:**
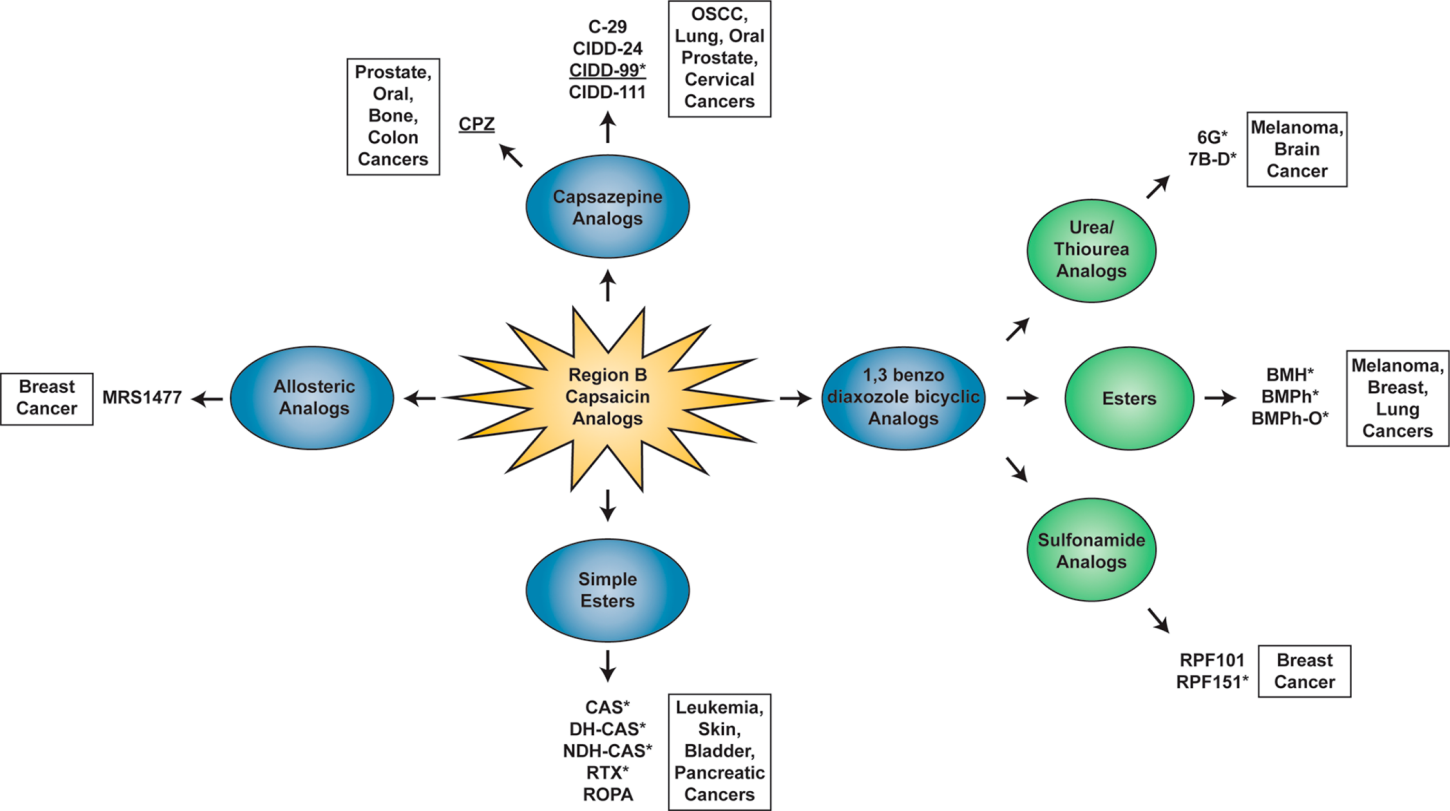
SAR studies of region B capsaicin analogs and their applications in human cancers. The analogs marked with * selectively suppress the growth of human cancer, sparing normal cells. The underlined analogs have been used to sensitize human cancer cells toward the growth-suppressive activity with chemotherapeutic drugs and radiation.

A majority of the studies exploring the growth-suppressive activity of region B capsaicin analogs have been conducted in cell culture models with only a few published reports investigating the anticancer activity of these compounds in animal model systems. Figure 9 summarizes the SAR studies of region B capsaicin analogs and their applications in human cancers. This portion of the perspective has been subdivided into two sections. The first section describes the anticancer activity of natural region B capsaicin analogs (Table 3). The second section summarizes the growth-suppressive activity of synthetic region B capsaicin analogs (Table 4). An interesting observation is that a substantial number of region B capsaicin analogs selectively induce apoptosis in human cancer cells and minimally impact the viability of normal cells. Such selectivity for cancer cells makes these drugs attractive candidates for cancer therapy. The molecular mechanisms underlying the growth-suppressive activity of region B capsaicin analogs include cell cycle arrest, alteration of mitochondrial function, generation of ROS and apoptosis. A few region B capsaicin analogs have been shown to possess antiangiogenic, antimigratory, and anti-invasive activity. Such observations suggest that region B capsaicin analogs abrogate the growth of the primary tumor as well as its metastasis to secondary organ sites.

**Table 3:** Anti-neoplastic Activity of Natural Region B Capsaicin Analogs

analog	type of cells	IC_50_ (in cell culture)	effect on normal cells	experimental models used	phenotypic effects	role of TRPV1	mechanism of action	ref
**2, 3**	HUVEC	ND		cell culture, *ex vivo* rat aortic rings model, mouse model of angiogenesis	inhibition of VEGF-induced cell proliferation, chemotaxis, permeability, and angiogenesis	ND	suppression of VEGF-induced Src kinase activity; phosphorylation of FAK, VE-cadherin; and inhibition of VEGF-induced endothelial cell–cell junctions	(105)
**4**	T-cell leukemia cells	Jurkat: 70 μM	no effect on the viability of normal T-cells	cell culture	induction of apoptosis; inhibition of cell proliferation	not *via* TRPV1 pathway	disruption of mitochondrial membrane potential; elevation of ROS levels; and activation of caspase-3	(69, 80)
**4**	skin cancer	ND	ND	two-stage mouse model of skin carcinogenesis	decrease in the number and volume of skin papilloma tumors	ND	ND	(69)
**5**	bladder cancer cells	T24: 21.4 μM	lower growth-inhibitory activity in normal urothelial cells	cell culture and athymic mouse model	cell cycle arrest and necrosis in bladder cancer cell lines; inhibition of tumor growth in athymic mice	not via TRPV1 pathway	disruption of mitochondrial membrane potential; elevation of ROS production	(111)
		5637: 19.9 μM						
**5**	cutaneous squamous cell carcinoma cells	ND	ND	cell culture	induction of apoptosis	not via TRPV1 pathway	inhibition of mitochondrial electron transport chain; upregulation of ROS	(116)
**5**	pancreatic cancer cells	ND	ND	cell culture	inhibition of cell viability and induction of apoptosis	not via TRPV1 pathway	blockage of mitochondrial electron transport chain; elevation of ROS	(115)
**5**	T-cell leukemia cells	Jurkat: 70 μM	ND	cell culture	induction of apoptosis in S-phase of the cell cycle	ND	disruption of mitochondrial membrane potential; and elevation of ROS levels	(114)
**5**	rat ileal epithelial cells	ND		cell culture	inhibition of cell proliferation	not via TRPV1 pathway	suppression of mitochondrial respiration; mitochondrial membrane depolarization; and inhibition of cyclin D1	(119)
**6**	rat ileal epithelial cells	ND	ND	cell culture	transient inhibition of cell proliferation	not via TRPV1 pathway	activation of PKC, activation of p21; transient inhibition of cyclin D1; and alteration of mitochondrial function	(119)

**Table 4:** Anti-neoplastic Activity of Synthetic Region B Capsaicin Analogs

analog	type of cancer	IC_50_ (in cell culture)	effect on normal cells	experimental models used	phenotypic effects	role of TRPV1	mechanism of action	ref
**7**	breast cancer	MCF-7: 3 μM	ND	cell culture	induction of apoptosis in cell culture, and no effect in mouse models	via the TRPV1 pathway	disruption of mitochondrial membrane potential; elevation of ROS levels; and activation of caspase-3, 9	(120)
		MDA-MB-231: 40.8 μM		NSG mouse xenograft model				
		BT-474: 10.6 μM						
**8**	melanoma	B16-F10: 87 μM	no effect on normal lung cells	cell culture	inhibition of cell viability	ND	ND	(83)
		SK-MEL-28: 85 μM						
**9**	lung cancer	H1299: 172 μM	no effect on normal lung cells	cell culture	inhibition of cell viability	ND	ND	(83)
**10**	melanoma lung cancer	B16-F10: 117 μM	no effect on normal lung cells	cell culture	inhibition of cell viability	ND	ND	(83)
		H1299: 187 μM						
**11**	breast, skin cancer	MCF-7: 32 μM	ND	cell culture and 3D spheroid model	inhibition of cell viability, cell cycle arrest (G2/M), and induction of apoptosis	ND	disruption of mitochondrial membrane potential; dysregulation of microtubule formation; and mitotic catastrophe	(84)
		MDA-MB-231: 14.2 μM						
		SK-MEL-28: 19.1 μM						
		Sbcl2: 17.5 μM						
		Mel-85: 15.7 μM						
**12**	breast cancer	MDA-MB-231: 87 μM	lower growth-inhibitory activity in normal breast epithelial cells	cell culture and athymic mouse model	inhibition of cell viability; cell cycle arrest (G1phase); anoikis; induction of apoptosis; and inhibition of breast tumor growth in athymic mice	not via the TRPV1 pathway	reduction of mitochondrial membrane potential; activation of TRAIL pathway; elevation of caspase3, ROS, and p21; and decrease in cyclin A, D1, D3, and BCl-2	(85)
**13**	melanoma and brain cancer	SK-MEL-25: 67.2 μM	no effect on normal human fibroblasts	cell culture and athymic mouse model	inhibition of cell viability; cell cycle arrest (G1phase); induction of apoptosis; and inhibition of melanoma growth in athymic mice	not via the TRPV1 pathway	activation of caspase-3 and decrease in BCl-xL	(88)
		A2058: 55.2 μM						
**14**–**16**	melanoma and brain cancer	SK-MEL-25: 67.2 μM	no effect on normal human fibroblasts	cell culture	inhibition of cell viability	ND	ND	(88)
		A2058: 55.2 μM						
		U87MG: 86.9 μM						
**17**	prostate cancer, oral cancer, bone cancer, and colon cancer	DU145:54 μM; IC_50_ was not determined for the other cancers	ND	cell culture and athymic mouse model	inhibition of cell viability, induction of apoptosis; inhibition of tumor prostate cancer growth in athymic mice; and inhibition of invasion	ND	increase in intracellular calcium; ER stress; elevation of ROS, JNK, and CHOP; and decrease of STAT3 and JAK	(121−125)
**18**	cervical cancer, oral cancer, lung cancer, and prostate cancer	HeLa: less than 5 μM	ND	cell culture and athymic mouse model (for Hela)	inhibition of cell viability and inhibition of HeLa tumor growth in athymic mice	not via the TRPV1 pathway	ND	(95)
		HSC:2 μM						
		H460: 42 μM						
		MDA-MB-231: 32 μM						
		PC-3: 5 μM						
**19**	cervical cancer, oral cancer, lung cancer, and prostate cancer	HeLa: < 5 μM	ND	cell culture and athymic mouse model (for Hela)	inhibition of cell viability and inhibition of HeLa tumor growth in athymic mice	via the TRPV1 pathway	ND	(95)
		H460: 23 μM						
		MDA-MB-231: 5 μM						
		PC-3: 13 μM						
**19, 20**	OSCC	CAL27: 20 μM	ND	cell culture and athymic mouse model	inhibition of cell viability, cell cycle arrest (S-phase); induction of apoptosis; and inhibition of OSCC tumor growth in athymic mice	ND	ND	(96)
		HSC3: 20 μM						
		SCC4: 30 μM						
		SCC9: 40 μM						
**21**	cervical cancer, lung cancer, and prostate cancer	HeLa: ∼ 5 μM	ND	cell culture	inhibition of cell viability	not via the TRPV1 pathway	ND	(96)
		H460:7.5 μM						
		MDA-MB-231: 2.5 μM						
		PC-3: 1 μM						
**21**	OSCC	CAL27: ∼ 5 μM	no effect on the growth of normal human keratinocytes at IC_50_ concentration	cell culture and athymic mouse model	inhibition of cell viability, cell cycle arrest (S-phase), and induction of apoptosis	not via the TRPV1 pathway	increase in intracellular calcium; ER stress; elevation of ROS, JNK, BiP, and CHOP; decrease of STAT3 and JAK; and mitochondrial depolarization	(96)
		HSC3: 1 μM						
		SCC4: 10 μM						
		SCC9: 1 μM						

### 9.1 Antineoplastic Activity of Natural Region B Capsaicin Analogs

#### 9.1.1 Capsiates

Among all of the known capsinoids, the growth-inhibitory activity of **4** has been most extensively studied in human cancer cell lines. Capsinoid **4** triggered robust apoptosis in Jurkat human T-cell leukemia cells which were treated with concentrations of **1** or **4** ranging from 1 to 200 μM) for 18 h,^69,80^ with the pro-apoptotic activity measured by flow cytometry. Capsaicin analog **4** was more potent in triggering apoptosis in Jurkat cells (IC_50_ value ∼70 μM) than **1**, IC_50_ value ∼128 μM, as summarized in Table 1.^69^ More detailed studies indicated that **4** triggered apoptosis in Jurkat cells via a TRPV1-independent mechanism. Moreover, **4** displayed robust chemopreventive activity in an *in vivo* two-stage model of mouse skin carcinogenesis,^69^ results that tempt speculation that **4** may provide protection against skin cancer.^69^ The growth suppressive activity of **4** in Jurkat cells was found to be due to a combination of cell cycle arrest (at S-phase) and apoptosis (Table 3). The treatment of Jurkat cells with **4** induced the activation of caspase-3, which led to downstream apoptosis in these cells. Another important mechanism underlying the apoptotic activity of **4** was its ability to alter the redox balance of Jurkat cells^69,80^ by disrupting mitochondrial membrane potential and elevating the levels of ROS. The ability of **4** to elevate ROS in Jurkat cells was greater than that of **1**. The free phenolic OH group in **4** was required for the pro-apoptotic activity of these compounds since the phenol methyl ether abolished the ability of **4** to elevate ROS and trigger cell death in Jurkat cells.^80^ An interesting observation was that capsinoids did not trigger any cell death in normal T-cells.^69,102^ Purified T-lymphocytes were activated using OKT-3 and then treated with **1** or **4** at a concentration of 100 μM for 3 days. Flow cytometry assays revealed that neither **1** or **4** triggered any apoptosis or proliferation in these activated T-cells.

Apart from directly inhibiting the growth of cancer cells, capsinoids also potently block tumor neovascularization (also called angiogenesis). Angiogenesis refers to the growth of new blood vessels from preexisting blood vessels and is required for physiological processes like wound-healing, menstruation, and embryogenesis. However, angiogenesis also plays a vital role in the growth of progression of human cancers, and the acquisition of an “angiogenic phenotype” is considered decisive for tumor progression.^103,104^ The recruitment of blood vessels is crucial for the sustained growth of the primary tumor mass as it allows oxygenation and nutrient perfusion of the tumor as well as removal of waste products. In addition, the vascular endothelial cells stimulate the proliferation of tumor cells in an autocrine and paracrine manner. The onset of increased angiogenesis coincides with the entry of tumor cells into circulation and facilitates distant metastasis. Several convergent studies have indicated that the transition from an *in situ* carcinoma to an invasive cancer must be accompanied by angiogenesis. Consequently, the inhibition of angiogenesis is considered to be one of the most promising strategies for the development of anticancer therapies.

Capsinoids **2** and **3** displayed robust antiangiogenic activity in cell culture and mouse model systems.^105^ Angiogenesis is a complex multistep process involving endothelial cell proliferation, chemotactic migration, invasion into blood capillaries, and differentiation into new blood vessels.^103^ Several lines of evidence show that angiogenic blood vessels are more permeable and “leaky” than normal blood vessels.^106^ The increased permeability of angiogenic blood vessels facilitates increased entry of growth factors and nutrients into the bloodstream, which in turn accelerates the progression of human tumors. Vascular endothelial growth factor (VEGF) is a potent pro-angiogenic growth factor which stimulates tumor angiogenesis by stimulating proliferation of endothelial cells and increasing the permeability (or leakiness) of angiogenic blood vessels.^107,108^ The treatment of human umbilical vein endothelial cells (HUVECs) with **2** and **3** (at a concentration of 25 μM) inhibited VEGF-induced cell proliferation and chemotaxis *in vitro*. The ability of **2** and **3** to inhibit VEGF-induced permeability of HUVECs was measured by the sucrose transport assay. In this experiment, the rate of transport of radiolabeled sucrose across a confluent monolayer of HUVECs was measured by a scintillation counter. The treatment of HUVEC’s with 50 ng/mL VEGF increased the rate of sucrose transport by 1.6-fold relative to untreated cells.^105^ The treatment of HUVECs with **2** and **3** at concentrations ranging from 1 to 25 μM abolished VEGF-induced elevation of the permeability of endothelial cells. The maximal antipermeability activity of **2** and **3** was observed at 25 μM.

The antiangiogenic activity of **2** and **3** was measured by the “Matrigel model assay” *in vitro*. The treatment of HUVECs with VEGF led to the formation of a dense network of capillary tubelike structures on Matrigel over 24 h.^105^ The addition of 25 μM of **2** and **3** along with VEGF blocked the formation of angiogenic capillary tubelike structures on Matrigel by 3.5-fold relative to endothelial cells treated with VEGF only. Similarly, concentrations of 25 μM **2** and **3** suppressed VEGF-induced angiogenic sprouting in the *ex vivo* rat aortic ring model of angiogenesis.^105^ The ability of **2** and **3** to block VEGF-induced angiogenesis *in vivo* was examined by the “Matrigel Plug assay” in C57BL6 mice. C57/BL6 mice were subcutaneously injected with Matrigel containing 100 ng VEGF in the presence or absence of 60 μg of **2** or **3**. The injected Matrigel rapidly formed a single, solid gel plug under the skin of mice (Table 3). After 1 week, the mice were euthanized and the Matrigel plugs were collected and immunostained with CD31, a blood vessel marker.^105^ The Matrigel plugs isolated from the VEGF-treated mice displayed an abundant number of CD31-stained cells. Co-treatment of **2** or **3** along with VEGF decreased the number of CD31-stained cells by 35-fold relative to mice treated with VEGF only (Table 1).

Immunoblotting experiments and kinase assays revealed that **2** and **3** at a concentration of 25 μM suppressed VEGF-induced angiogenesis via direct suppression of Src kinase activity and phosphorylation of its downstream substrates, including p125^FAK^ and VE-cadherin, in human endothelial cells.^105^ Both compounds also blocked VEGF-induced endothelial permeability and loss of VE-cadherin-facilitated cell–cell junctions, at a concentration of 25 μM (Table 3). Molecular modeling and docking experiments suggested that **2** can directly bind to the ATP-binding pocket of Src kinase.^105^ However, no binding assays were performed to confirm such direct interaction between Src kinase and **2**.

Capsaicin analog **2** did not have any detrimental impact in C57BL6 mice (which were used for the *in vivo* Matrigel plug experiments).^105^ The fact that **2** does not kill normal cells was subsequently confirmed in a study that examined the effect of **2** on normal hepatocytes in mice (Table 3). Daily oral administration of **2** at a dose of 60 mg/kg bodyweight for 30 days did not induce any injury to the liver.^109^ Histological analysis of harvested livers at the end of the experiment revealed that **2** did not induce apoptosis, and the levels of the liver enzymes AST, ALT, and alkaline phosphatase remained unchanged.

#### 9.1.2 Resiniferatoxin (RTX) and ROPA

The heterocyclic vanilloid compound **5** is about 500–1000 times more pungent than **1** which may be explained by its high affinity for the TRPV1 receptor.^110^ The growth suppressive activity of **5** has been explored in multiple human cancer cell lines, with the results compiled in Table 3. The data generated show that **5** decreased the viability of T24 and 5637 human bladder cancer cells at concentrations ranging from 10 to 100 μM at 24 h postdose.^111^ Administration of **5** once every 3 days over a period of 3 weeks at a concentration of 10 μM robustly decreased the growth rate of T24 human bladder cancer tumors xenografted in athymic mice.^111^ The treatment of mice with **5** did not result in any gross toxicity, the weights of the mice were not altered, and none of the mice died. Also, no inflammatory infiltrates were detected in the epithelial and subepithelial tissues surrounding the tumors in mice treated with **5**. The growth-inhibitory effects of **5** were found to be the result of a combination of cell cycle arrest and necrosis. No evidence of apoptosis was found in bladder cancer cells *in vitro* or *in vivo* treated with **5**. It must be remembered that T24 cells express the TRPV1 receptor, but 5637 cells are TRPV1-negative.^111^ Consequently, the fact that the IC_50_ values of **5** in T24 cells (IC_50_ ∼ 21.4 μM) and 5637 cells (IC_50_ = 19.9 μM) are almost identical suggests that the growth-suppressive activity of **5** is mediated *via* TRPV1-independent mechanisms (Table 3). In support of this hypothesis, treatment of T24 cells with the TRPV1 antagonist 5′-iodoresiniferatoxin did not impact the growth-inhibitory activity of **5**. Such observations support the notion that the TRPV1 receptor is not involved in the growth-suppressive effects of **5** in bladder cancer cells. The ability of **5** to induce necrotic cell death was attributed to its ability to alter mitochondrial function, increase the ADP/ATP ratio, and elevate ROS production in human bladder cancer cells.^111^ Normal human urothelial cells (NUCCs) show reduced sensitivity to the growth-inhibitory activity of **5** compared to bladder cancer cells (Table 3). The research paper did not provide any insights for why **5** triggered a lower magnitude of apoptosis in NUCC’s. Several congruent studies have confirmed that mitochondrial function is altered in human cancer cells as compared to normal cells. For example, the mitochondria of neoplastic cells are more hyperpolarized than normal cells.^112^ Similarly, the levels of ROS and activity of ROS-sensitive pathways is lower in normal cells than cancer cells.^113^ Taken together, these two observations may explain the reduced sensitivity of NUCCs toward the effects of **5**.

Capsaicin analog **5** was found to trigger apoptosis of Jurkat T-cell leukemia cells;^114^ however, the concentration of **5** used in this experiment was extremely high (∼10 M). Such findings suggest that **5** is not a viable anticancer drug for the treatment of T-cell leukemia. The treatment of Jurkat cells with 10 M **5** induced programmed cell death during the S-phase, and a small amount of apoptosis was also observed in cells in the G0/G1 phase.^114^ The pro-apoptotic effects of **5** were dependent on its ability to disrupt mitochondrial membrane potential and elevate ROS in Jurkat cells (Table 3). However, a caveat of this study was that no explanation was provided about why such a high concentration of **5** was required to induce programmed cell death in Jurkat cells.

**Figure 10:**
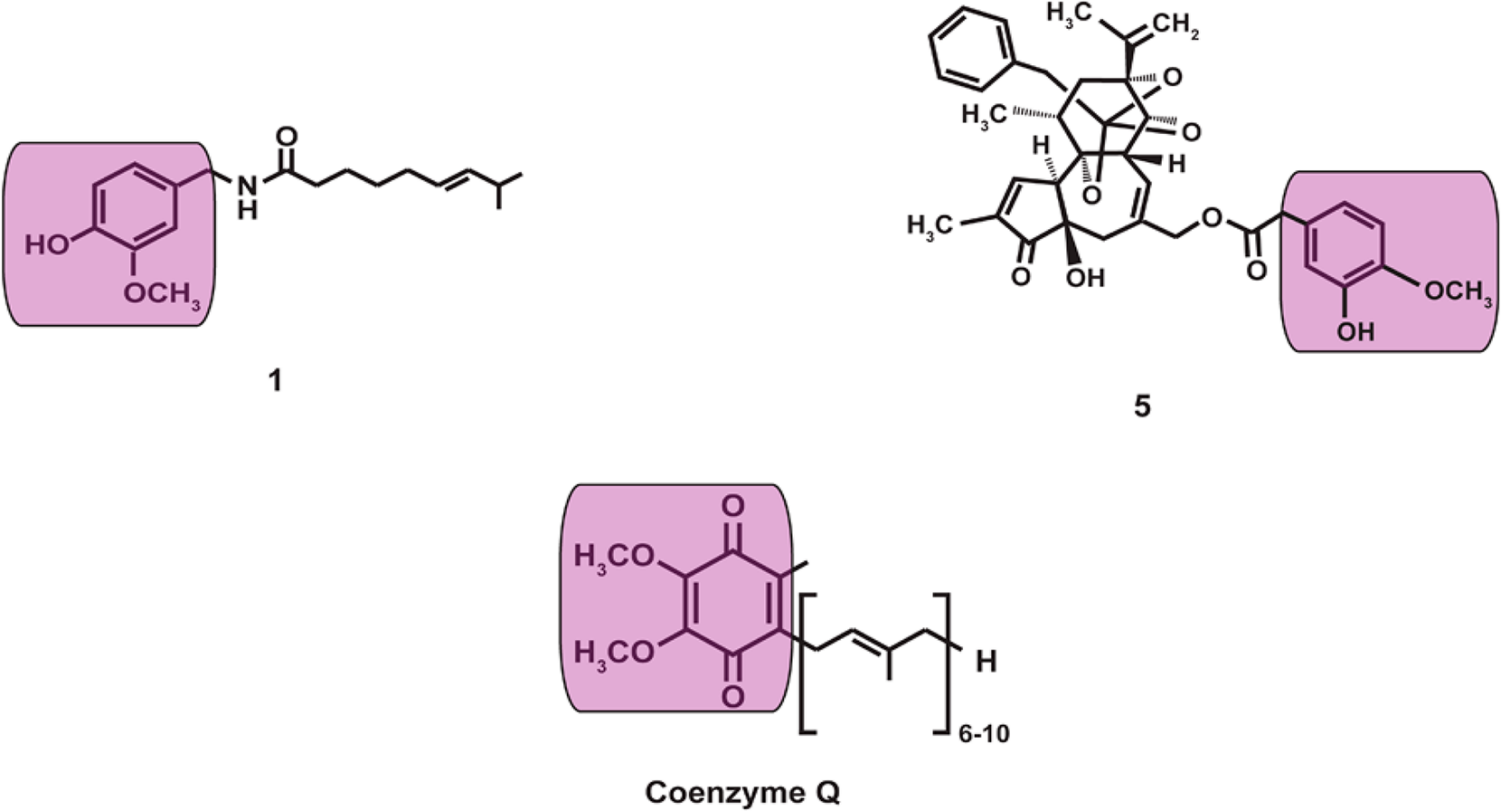
A comparison of the structures of **1** and **5** with coenzyme Q. The area in purple shows the regions of similarity in the three compounds.

The pro-apoptotic activity of **5** was compared to **1** by treating COLO 16 and SRB-12 human cutaneous squamous cell cancer (SCC) cells with varying concentrations of both compounds (ranging from 0 to 200 μM) over a period of 24 h. It was observed that 10 μM of **5** induced a similar magnitude of apoptosis (in COLO 16 cells) as 100 μM of **1**. Similarly, the treatment of 10 μM of **5** triggered robust apoptosis in MIA PaCa-2 and Capan-1 human pancreatic cancer cells over 24 h.^115^ It was observed that the programmed cell death induced by **5** (in human pancreatic cancer cells and SCCs) was mediated by its ability to block the mitochondrial electron transport chain (Table 3). Both **1** and **5** have been shown to be potent inhibitors of complex I of the mitochondrial electron transport chain.^20^ The vanillyl moiety of **1** and **5** (indicated by the purple color in Figure 10) is structurally similar to the cyclic portion of coenzyme Q, which explains the fact that these compounds act as coenzyme Q antagonists.^116^ Similarly, the hydrophobic domains (attached to the vanillyl moieties) **1** and **5** correspond to the isoprenoid chain of coenzyme Q. The relative hydrophobicity of these domains has been thought to contribute to their activity as coenzyme Q inhibitors.^116^ This fact may explain why **5** was as effective as **1** in inducing apoptosis (and inhibiting mitochondrial respiration), even when it was used at a 10-fold lower concentration than **1** in human SCC’s.

The protein coenzyme Q is a cofactor in the electron transport chain and scavenges reactive oxygen species to protect tissues from ROS-mediated damage.^117,118^ The inhibition of coenzyme Q results in the leakage of electrons to oxygen, producing the superoxide anion, hydrogen peroxide, and hydroxyl radical, which elevates reactive oxygen species thereby leading to apoptosis of SCC’s and pancreatic cancer cells.^116^ Finally, the pro-apoptotic activity of **5** in both SCC’s and pancreatic cells was independent of the TRPV1 pathway.

Apart from being an effective anticancer agent on its own, studies have evaluated if **5** could improve the growth-inhibitory activity of the chemotherapy drugs 5-fluorouracil and gemcitabine (Table 3); however, no additive/synergistic interaction was found in both pancreatic cancer cell lines.^115^

Compound **6** is a hydrolysis product of **5** (Figure 2), and the growth-suppressive activities of the two compounds has been compared using IEC-18 rat ileal epithelial cells.^119^ The treatment of IEC-18 cells with 7.5 μM **6** induced a transient PKC-dependent cell cycle arrest in G1 phase over 6 h which was reversed by 12 h (Table 1). The antiproliferative activity of **6** was found to be mediated by a protein kinase C (PKC)-dependent pathway. PKC are a family of calcium-activated, phospholipid-dependent serine-threonine kinases which function as a cellular target for phorbol esters. The PKC pathway plays a vital role in the proliferation of the self-renewing intestinal epithelium. The unique architecture of this tissue, with its well-defined regions of cell proliferation, differentiation, mature function, and senescence, correlates with changes in the expression and activation of PKC isozymes. Morphological and biochemical assays have revealed that the PKC isozymes are activated within intestinal crypts (where the cells are quiescent), suggesting that the PKC pathway is involved in negative regulation of cell growth in this system. The treatment of IEC-18 rat ileal epithelial cells with **6** have shown that it causes a robust activation of PKC enzymes. However, ROPA-induced activation of PKC is a transient phenomenon which lasts up to 12 h after drug treatment (Table 3). After 12 h, **6** produces a downregulation of PKC, which explains the reversal of cell cycle arrest observed by ∼12 h of treatment.

The cell-cycle inhibitory effects of **6** were also correlated with a decrease in cyclin D1 levels and concomitant upregulation of p21 expression.^119^ Similar to its effect on the cell cycle profile of IEC-18 cells, the downregulation of cyclin D1 by **6** was transient, with levels returning to normal between 12 and 16 h (Table 3). In contrast, concentrations of **5** ranging from 5 to 10 μM induced prolonged G0/G1 arrest in IEC-18 cells. Cell cycle analysis revealed that **5**-induced G0 arrest was evident by 6 h and lasted up to 24 h. In addition, **5** triggered sustained a long-term decrease of cyclin D1 levels in IEC-18 cells. However, unlike **6**, **5** did not exert any effect on p21 levels in IEC-18 cells.^119^

No involvement of PKC was observed in **5**-induced cell cycle arrest. A remarkable observation was that the growth-inhibitory activity of **6** as well as **5** was found to be independent of the TRPV1 receptor family.^119^ The growth-suppressive activity of **5** and **6** were mediated by diverse mechanisms, including suppression of mitochondrial respiration, mitochondrial depolarization, and generation of ROS, which led to downstream sustained long-term downregulation of cyclin D1 expression (Table 3). A paradoxical observation was that **5** had a meager impact on the viability of NUCCs,^111^ whereas it induced prolonged cell cycle arrest within G0 phase in IEC-18 rat ileal epithelial cells.^119^ Such divergent results can be explained by the fact that the IEC-18 is an immature epithelial cell line derived from rat intestinal crypt, and therefore, its growth characteristics cannot be compared to normal primary adult epithelial cells.^119^ Additionally, there may be subtle species–specific molecular differences between rat and human cell lines that could underlie the varying response of **5** between IEC-18 and NUCCs.

#### 9.1.3 Allosteric TRPV1 Modulators

The dihydropyridine-based capsaicin analog **7** is a positive allosteric modulator of TRPV1 (Figure 2). Treatment of MCF-7, MDA-MB-231, and BT-474 human breast cancer cells with 2 μM **7** potently triggered programmed cell death over 72 h (Table 4). An important fact to note was that **7** only exerted its pro-apoptotic activity on cells which were pretreated with **1** based on the rationale of activating the TRPV1 receptor. The central hypothesis pursued in these studies was that the TRPV1 receptor was activated by endogenous cellular factors in human breast cancers. Therefore, the TRPV1 receptor on these cells was activated by pretreatment with **1** and the cells exposed to varying concentrations of **7**. Under this experimental circumstance, **7** potently blocked the growth of capsaicin-treated human breast cancer cell lines *in vitro* in a fashion that was dependent on cellular apoptosis.^120^ The pro-apoptotic effects of **7** required the TRPV1 receptor and induced downstream activation of the caspase-3 and caspase-9 pathway. Furthermore, **7** induced mitochondrial depolarization, disruption of mitochondrial membrane potential, and a robust increase in ROS levels in MCF-7 cells.^120^ Notably, **7** did not affect the viability of normal breast epithelial cells.^120^

Subsequently, the antitumor activity of **7** was tested *in vivo* using immunodeficient NSG mouse models xenografted with human breast cancer tumors. The genetic background of the NSG mice involves a combination of severe combined immune deficiency (*Scid*) and the absence of the IL2 receptor common gamma chain. Since these mice lack a functional immune system, human breast cancer cells injected subcutaneously into these mice develop into tumors. In this setting, the administration of **7** at a dose of 10 mg/kg bodyweight, injected intraperitonealy twice a week, had no impact on the growth rate of xenotransplanted MCF-7 tumors. However, an important fact to note is that the tumor-bearing NSG mice were not pretreated with **1** before the administration of **7**. Presumably, it was assumed that endogenous cellular factors would have activated the TRPV1 receptor in these breast tumors *in vivo*. Another possibility may be that the pharmacokinetic properties of **7** are not suitable to display antitumor activity *in vivo*. Although, **7** did not show significant anticancer activity when administered alone, it may have applications when combined with low doses of **1** or standard-of-care chemotherapeutic drugs.

#### 9.1.4 Capsaicin Analogs Containing a 1,3-Benzodioxole Structural Motif

The BMH series of capsaicin analogs contain the 1,3-benzodioxole structural motif in region A and an ester group in region B (Figure 2).^83^ Three of these compounds, **8**, **9**, and **10**, were tested for their ability to decrease the viability of a diverse panel of human cancer cells over a period of 24 h (Table 4). The cell lines tested included murine melanoma (B16-F10), human melanoma (SK-MEL-28), human lung cancer cells (H1299 and H460), and human breast cancer cell lines (SK-BR-3 and MDA-MB-231). The growth-suppressive activity of **8**−**10** were also evaluated in MRC-5 normal human lung cells.^83^ MTT assays revealed that the compound **8** was the most potent in suppressing the viability of B16-F10 murine melanoma cells (IC_50_ ∼ 130 μM) and SK-MEL-28 human melanoma cells (IC_50_ ∼ 85 μM). Compounds **9** and **10** displayed meager growth-inhibitory activity in all the above human-mentioned cancer cell lines. Compound **9** decreased the viability of H1299 cells at rather high concentrations, IC_50_ ∼ 172 μM.^83^ Compound **10** displayed modest growth-suppressive activity in B16-F10 human lung cancer cells, IC_50_ ∼ 87 μM. A notable observation was that the growth-suppressive activity of **9** and **10** was greater than that of **1** (IC_50_ for capsaicin ≥200 μM in H1299 cells; IC_50_ for capsaicin in B16-F10 cells = 117 μM) in murine melanoma and human lung cancer cells. None of these analogs had any impact on the growth of normal lung cells.^83^

Compounds **11** and **12** are alkyl/aryl sulfonamide-based analogs of **1**.^84,85^ The growth-suppressive activity of **11** (IC_50_ ∼ 32 μM) was about 2-fold higher than **1** (IC_50_ ∼ 53 μM) in MCF-7 cells over 48 h (Table 4). The treatment of MCF-7 cells with 32 μM of **11** triggered morphological changes including cell shrinkage, apoptosis, and pyknosis. Furthermore, **11** suppressed the growth of three-dimensional spheroid cultures of MCF-7 cells (grown on Matrigel) at a concentration of 32 μM. The growth-suppressive activity of **11** could be attributed to a combination of cell cycle arrest at the G2/M phase and apoptosis.^84^ In addition, the treatment of human breast cancer cells with 32 μM of **11** caused disruption of mitochondrial membrane potential, dysregulation of microtubule formation, and mitotic catastrophe to induce cell cycle arrest and apoptosis.

The chemical modification of **11** produced **12** (Figure 2) which offered better chemical stability and higher aqueous solubility properties.^85^ MTT assays revealed that the growth suppressive activity of **12** (IC_50_ ∼ 87 μM) was better than **1** (IC_50_ ∼ 120 μM) in MDA-MB-231 human breast cancer cells.^85^ Compound **12** showed lower growth suppressive activity in MCF10A normal human breast epithelial cells (IC_50_ ∼ 198 μM) relative to MDA-MB-231 human breast cancer cells (Table 4). A caveat of this research paper is that the growth suppressive activity of **12** was not compared with **11**. Another drawback of these experiments was the absence of statistical analysis of the data. Such facts make it impossible to infer whether if the IC_50_ values obtained in the MTT assays were significantly different from each other.

The mechanism of action of **12** was different from **11** in human breast cancer cells, with the former inducing cell cycle arrest at the S-phase (at a concentration of 87 μM) with concomitant decrease in cyclin A, D1, and D3 levels (Table 4). Furthermore, **12** induced apoptosis in MDA-MB-231 cells *via* downregulation of BCl-2, an increase in the expression of p21, caspase3, phospho-BAD, and ROS (at a concentration of 87 μM), and a reduction of mitochondrial membrane potential and activation of the TRAIL pathway.^85^ Treatment of MDA-MB-231 human breast cancer cells with 87 μM of **12** caused cytoskeletal remodeling and anoikis. Most remarkably, the growth-suppressive activity of **12** was independent of the TRPV1 receptor. The antineoplastic activity of **12** was evaluated *in vivo* in athymic mice xenotransplanted with MDA-MB-231 human breast cancer cells. The tumor-bearing mice were administered **12** at a dose of 70 mg/kg bodyweight given daily by intraperitoneal injection over 25 days (Table 4). Compound **12** induced a 3-fold decrease in tumor volume relative to the control group and was not associated with any gross toxicity or discomfort in the mice.^85^

The growth-suppressive activities of **13**–**16**, urea, and thiourea analogs of **1** were tested in a panel of human cancer cell lines^88^ that included A2058, SK-MEL-25 (human melanoma), and U87MG (human glioblastoma). Compound **13** (IC_50_ = 50–70 μM) was almost 2-fold more potent than **1** (IC_50_ > 100 μM) in decreasing the viability of human melanoma cells. In contrast, the growth suppressive activity of **13** in human glioblastoma cells (IC_50_ ∼ 87 μM) was modestly greater than **1** (IC_50_ > 100 μM). The growth-inhibitory activity of **14**–**16** (IC_50_ = 85–98 μM) was lower than **13** in human melanoma cells (Table 4). In contrast, the growth suppressive activity of **13**–**16** was similar in human glioblastoma cells (IC_50_ = 87–98 μM) while none had any impact on the growth of normal human fibroblasts.^88^ Of this series of compounds, the growth-inhibitory activity of **13** in human melanoma cells was partially dependent on TRPV1. The ability of **13** to decrease the viability of human melanoma cells was due to a mixture of cell cycle arrest (G0/G1 phase) and apoptosis (Table 4). Immunoblotting experiments revealed that the B-Raf/MAP kinase pathway, especially MEK1, was the cellular target of **13**. The pro-apoptotic activity of **13** was mediated by the caspase-3 pathway and correlated with a decrease in Bcl-xL expression.^88^ Compound **13** had a higher log *P* value than **1** which indicated that **13** had higher lipophilicity relative to **1**. Based on these log *P* values, it was inferred that **13** would have higher membrane permeability and bioavailability compared to **1**. However, no experiments were conducted in animal models to confirm these findings.

#### 9.1.5 Capsazepine and Its Analogs

Compound **17** is a benzazepine compound which functions as a potent antagonist of TRPV1,^93,121^ and several convergent studies indicate that it displays robust growth-suppressive activity in human prostate cancer, oral cancer, colorectal cancer, and osteosarcoma cell lines.^121^ In a model of prostate cancer conducted in athymic mice, intraperitoneal administration of **17** at doses of 1 and 5 mg/kg three times a week for 25 days resulted in decreased tumor volume by 2–2.5-fold relative to the control group of mice.^122^ Apart from prostate cancer, **17** displayed robust antitumor activity in human oral squamous cell carcinoma (OSCC) *in vivo*.^123^ The intratumoral injection of **17** administered at a dose of 20 μg on alternate days decreased the growth rate of SCC-25 human OSCC tumors implanted in athymic mice by about 1.5-fold relative to vehicle-treated tumors (Table 4). A similar result was observed when **17** was administered to athymic mice bearing SCC4 human OSCC tumors at a dose of 40 μg injected directly into the tumor on alternate days.^123^ An innovative feature of this study was that the OSCC (called HSC3) cell line was developed from a cancer patient that was injected subcutaneously to athymic mice to generate patient-derived human OSCC tumors. Since the HSC3 cell line was isolated from a patient, it more closely reflects the pathophysiology of OSCC tumors in the clinic. Injection of **17** directly into HSC3 tumors implanted in athymic mice at a dose of 40 μg given on alternate days robustly decreased the growth rate by approximately 5-fold relative to vehicle-treated mice.^123^ The weights of mice were similar between the vehicle-treated group and the capsazepine-treated group, indicating that the administration of **17** was devoid of overt toxicity in these mice. There was no effect of **17** on the growth of nonmalignant tissues in these tumor-bearing athymic mice.^123^ The administration of **17** to athymic mice implanted with intrafemoral breast cancer tumors led to a decrease of cancer-related bone pain, relative to vehicle-treated mice (Table 4). The pain-relieving activity of **17** in mice was evaluated by behavioral testing and measurement of the ability of the tumor-bearing paw to support weight.^124^ Such analgesic activity of **17** was mediated by a TRPV1-dependent pathway.^121,125^

The primary mechanism underlying the growth-suppressive activity of **17** is its ability to trigger programmed cell death in human OSCC, prostate cancer, and oral cancer cells; however, **17** also targeted metastasis-related pathways by blocking the invasion of human prostate cancer cells.^121,122^ The pro-apoptotic activity of **17** was independent of the TRPV1 receptor, and programmed cell death appeared to be induced by multiple mechanisms, including an increase of intracellular calcium leading to endoplasmic reticulum stress, inhibition of the JAK/STAT3 pathway and elevation of ROS causing the activation of the JNK and CHOP pathways.^121^ Apart from being administered as a single agent, **17** could be combined with standard radiation therapy to robustly decrease the growth rate of human lung cancer cells.^126^ A549 human lung cancer cells were treated with 10 μM of **17** for 30 min and then irradiated with γ radiation at a dose of 2Gy for another 30 min. Flow cytometry assays revealed that the combination of **17** and gamma irradiation decreased the survival of A549 cells by 1.5-fold relative to **17** given alone. The combinatorial activity of **17** and γ radiation was mediated by the DNA damage pathways.^126^ Immunofluorescence assays showed that A549 human lung cancer cells treated with only **17** resulted in negligible effect on the expression of the DNA damage markers γH2AX and p53 binding protein-1. The combination of **17** with γ radiation led to a 6-fold increase in the expression of γH2AX, while the levels of p53 binding protein-1 were elevated by about 8-fold relative to treatment with **17** alone. A caveat of these studies is that the treatment of A549 cells with γ radiation alone decreased the survival of A549 cells by a greater magnitude than **17** combined with γ radiation.^126^ Similarly, the expression of γH2AX and p53 binding protein-1 in irradiated A549 cells was higher than the combination of γ radiation and **17**. Such observations make it difficult to interpret the clinical relevance of the combination therapy involving **17** and γ radiation.

SAR studies involving **17** resulted in the synthesis of almost 30 synthetic analogs which were screened for growth-inhibitory activity using HeLa human ovarian carcinoma cells.^95^ MTT assays revealed that four of these compounds, C-29 (**18**), CIDD-24 (**19**), CIDD-111 (**20**), and CIDD-99 (**21**), displayed the maximal decrease in cell viability of the HeLa cells over 24 h. The growth suppressive activities of **18**, **19**, and **21** were similar to each other (IC_50_ < 5 μM) in HeLa cells. However, the growth-inhibitory activity of **20** (IC_50_ ∼ 25 μM) was lower than **18**, **19**, and **21**. On the basis of these results, **18** and **21** were selected as “hit compounds” to be used in follow-up experiments. The initial experiments examined the effect of **18** and **21** on the viability of H460 (human lung cancer cells), MDA-MB-231 (human breast cancer cell line), PC-3 (human colon carcinoma cells), and HSC-3 [human oral squamous cell carcinoma (OSCC)] cells (Table 5). Out of these cell lines, the HSC-3 cells were directly established from an OSCC tumor resected from a cancer patient.^95^

**Table 5:** Growth-Inhibitory Activity of **18** and **21** in Human Cancer Cell Lines

cell line	type of assay	duration of assay	type of cancer cells	phenotypic effect	compound 18: IC_50_ value (μM)	compound 21: IC_50_ value (μM)
HSC3	MTS cell viability assay	24 h	patient-derived human OSCC cells	inhibition of cell viability	20	2
H460	MTS cell viability assay	24 h	human lung cancer cells	inhibition of cell viability	23	42
MDA-MB-231	MTS cell viability assay	24 h	human breast cancer cells	inhibition of cell viability	5	32
PC3	MTS cell viability assay	24 h	human colon cancer cells	inhibition of cell viability	13	2.5

The next series of experiments evaluated the antitumor activity of **18** and **21** in athymic mice models that had been subcutaneously injected with HeLa human cervical cancer cells.^95^ After the tumors reached a threshold value of 170 mm^3^, the mice were randomized into three groups, comprising of 4 mice each (Table 6). Compound **21** displayed better antitumor activity toward human cervical carcinoma tumors *in vivo* than **18**. There were no observable toxic effects of **18** and **21** in the athymic mice and no adverse effects on adjoining normal nonmalignant tissues. The compounds did not exhibit detrimental effects on mobility, motor functions, neurological, and respiratory function.^95^ Lastly, **18** and **21** did have any impact on the weights of mice.

**Table 6:** Anti-tumor Activity of **18** and **21** in Human Cervical Cancer Tumors *in Vivo*

group	type of treatment	effect on size of tumor after 14 days	mean tumor volume at the end of the study
1	vehicle	no decrease in tumor size	562 cm^3^
2	**18** (at a dose of 40 μg injected directly into the tumor every day for 14 days)	tumor size decreased 1.5-fold relative to group 1	351 cm^3^
3	**21** (at a dose of 40 μg injected directly into the tumor every day for 14 days)	tumor size decreased 2-fold relative to group 1	283 cm^3^

Compounds **17**, **19**, **20**, and **21** were evaluated for their growth-inhibitory activity in a panel of four human OSCC cell lines, namely HSC3, CAL27, SCC4, and SCC9.^96^ In all these cell lines, **21** displayed the most potent growth suppressive activity (IC_50_ = 1–10 μM) followed by **19** (IC_50_ = 2.5–40 μM) while the potency of **20** was the lowest (IC_50_ = 20–30 μM). All three compounds exhibited only meager growth-inhibitory activity toward OKT normal human keratinocytes and had no impact on cell viability at concentrations up to 7.5 μM (Table 4). Above 7.5 μM, these compounds decreased the viability of OKT cells by 20%. Subsequently, the antitumor activity of these compounds *in vivo* was compared with **17** in athymic mice models.^96^ The human OSCC cell line CAL27 was injected subcutaneously in the left flank of athymic mice. After the tumor volume reached 100 mm^3^, the mice were randomized into five groups, comprising of 5 mice each. The schema and the results of the study are summarized in Table 7. The antitumor activity of the compounds was ranked as **21** > **19** > **17** > **20** in CAL27 tumor-bearing mouse over a period of 4 weeks.^96^

**Table 7:** Anti-tumor Activity of **19–21** in Human OSCC Tumors *in Vivo*

group	type of treatment	effect on size of tumor at the end of 4 weeks	mean tumor volume at the end of the study
1	vehicle	no decrease in tumor size	541 cm^3^
2	**17** (at a dose of 120 μg injected directly into the tumor every day for 4 weeks)	tumor size decreased 2-fold relative to group 1	269 cm^3^
2	**19** (at a dose of 120 μg injected directly into the tumor every day for 4 weeks)	tumor size decreased 2.6-fold relative to group 1	211 cm^3^
3	**20** (at a dose of 120 μg injected directly into the tumor every alternate day for 4 weeks)	tumor decreased 1.6-fold relative to group 1	134 mm^3^
4	**21** (at a dose of 120 μg injected directly into the tumor every alternate day for 4 weeks)	tumor decreased 4-fold relative to group 1	333 mm^3^

In an independent experiment conducted using an athymic mice model, **21** was administered via intraperitoneal injection on alternate days to animals injected under the flank skin with CAL27 human OSCC cells.^96^ After the tumor grew to a volume of 100 mm^3^, the mice were randomized into two groups. The mice in the treatment group were administered **21** (at a dose of 12 mg/kg bodyweight) injected intraperitonealy on alternate days for a period of 4 weeks. At the end of the 4 weeks the tumors were harvested and the volumes measured. The administration of **21** decreased tumor volume by 7.3-fold when compared to the vehicle-treated control mice.^96^ In both athymic mouse experiments, **17** and **19**–**21** displayed no overt toxicity toward the mice, which is concordant with the finding that **21** did not impact the viability of OKT normal keratinocytes at its IC_50_ value (∼5 μM in CAL27 cells *in vitro*). The administration of **21** to athymic mice bearing CAL27 tumors did not affect the morphology of nonmalignant adjoining the tumor, and the compound did not trigger erythema, swelling, or ulceration in any tissue.^96^

In addition to its antitumor activity as a single agent, **21** was found to potentiate the growth-suppressive activity in CAL-27 cells human OSCC cells of standard-of-care chemotherapy drugs like cisplatin, gefitinib, and radiation.^96^ The combination of **21** (at a concentration 500 μM) and cisplatin (at a concentration of 25 μM) decreased the viability of human OSCC CAL27 cells^96^ by over 95% over a period of 24 h. In contrast, treatment of the cells with 25 μM cisplatin alone decreased the viability by 50% while exposure to 500 μM **21** alone decreased the cell viability by about 20%.^96^ Similarly, the combination of the epidermal growth factor receptor (EGFR) inhibitor gefitinib (at a concentration of 100 nM) with **21** (at a concentration 500 μM) suppressed the viability of CAL27 cells by ∼80%, which was higher than 100 nM gefitinib alone (decrease of cell viability was about 50%) and 500 μM **21** alone (decrease of cell viability was about 20%). Although the interaction between **21** and the chemotherapy drugs cisplatin and gefitinib was claimed to be synergistic, no statistical analyses were performed to confirm these effects. The nature of the interaction between two drugs given in combination can be determined by the Chou-Talalay isobologram analysis.^127,128^ The isobologram analysis yields a factor designated as “Combination Index (CI). If the CI is less than 1, the interaction between the two drugs is deemed synergistic. If the CI = 1, the interaction between the two drugs is said to be additive.^127,128^ In the absence of performing the Chou-Talalay statistical analysis, it is impossible to determine whether the interaction between the two drugs is additive/synergistic.

The pain-relieving activity of **1** is mediated by the TRPV1 receptor (also called the capsaicin receptor) on target cells where it acts as a potent agonist of the nonselective tetrameric cation channel receptor.^35^ The transmembrane domain of TRPV1 contains six transmembrane helices and exhibits structural features similar to voltage-gated potassium channels.^129^ The binding of capsaicin to the TRPV1 receptor induces the flow of calcium ions, thereby elevating the levels of intracellular calcium in target cells.^35^ In contrast, **17** is known to be a potent TRPV1 antagonist, and SAR studies of the pharmacophore of **17** led to the synthesis of **18**–**21**. An important question is whether the bioactivity of **18**–**21** requires the TRPV1 pathway.^96^ Compound **18** was found to be a potent agonist of the TRPV1 receptor in Chinese hamster ovary (CHO) cells overexpressing TRPV1 (hereby referred to as CHO-TRPV1 cells), with a 1 μM concentration of **18** elevating the levels of intracellular calcium in these cells.^96^ The pungent properties of **18** were evaluated by the “eye–wipe test” in rats.^96^ In this model, a drop of the drug in a solution of sterile saline was applied to one eye of the rat. The amount of time spent in closing the affected eye was recorded for a period of 4 min. The administration of **1** at a dose of 0.01% weight by volume in sterile saline to rats increased the orofacial pain levels (as determined by the eye-wipe test) by 1.8-fold relative to vehicle-treated control groups.^96^ Compound **18** was less pungent than 1 and increased the orofacial pain levels by 1.2-fold relative to vehicle-treated control groups. In contrast, compounds **19**–**21** did not have any impact on TRPV1-induced upregulation of calcium levels or on pain-sensation in rat models suggesting that the biological activity of **19**–**21** was independent of the TRPV1 pathway.^96^

It may appear paradoxical that **17** is a TRPV1 antagonist; however, the capsazepine analog **18** is a TRPV1 agonist. Pharmacological studies on several types of bioactive small molecules show that receptor selective agonist/antagonist properties may be conferred by optimizing the side-chain structure and the stereochemistry of functional groups on the molecule. An example is the fact that the scaffold of the neuropeptide somatostatin has been extensively used to obtain antagonists targeting a variety of receptors.^130,131^ The somatostatin backbone has been used to generate oxytocin antagonists, selective opioid antagonists with no residual somatostatin bioactivity, and neuromedin B antagonist.

Compounds **19**–**21** triggered cell cycle arrest in the S-phase and apoptosis in OSCC cells. The pro-apoptotic effects of **19**–**21** were mediated by elevation of ROS, an increase in intracellular calcium, induction of endoplasmic reticulum stress (as evidenced by rise of CHOP and BiP levels), and alteration of mitochondrial function.^96^ Together, these studies suggest that **17** and its analogs may be promising agents for the therapy of many types of human cancers.

## 10 Conclusions and Future Directions

The vanilloid phytochemical **1** displays potent antineoplastic activity in multiple human cancers^20,23,132^ and can improve the therapeutic efficacy of chemotherapeutic drugs and radiation therapy when used in combination.^26−28^ Despite such promising applications, the development of **1** as a clinically useful anticancer drug has been limited by its unpleasant side effects. The administration of **1** causes skin redness, hyperalgesia, nausea, intense tearing in the eyes, conjunctivitis, blepharospasm (sustained, forced, and involuntary closing of the eyelids), vomiting, abdominal pain, stomach cramps, bronchospasm, and burning diarrhea in patients.^133−135^ Clinical trials exploring the pain-relieving activity of **1** have shown that such side effects have resulted in patients discontinuing use of the drug. An advantage of several region B capsaicin analogs like **2**, **3**, and **4** is that they are nonpungent^4^ as are the synthetic capsaicin region B analogs like **12** which have low pungency properties.^85^ Therefore, these capsaicin-mimetics may have potential applications in cancer therapy. On the other hand, **5** is approximately 500- to 1000-fold more pungent than **1**(110) that, despite its high irritant properties, has emerged as a promising agent to control terminal cancer pain in patients.^64^ It is hoped that future studies may investigate the therapeutic potential of **5** in cancer therapy. Although **5** is extremely pungent, it could be delivered to patients as sustained release formulations like liposomes or nanoparticles to modulate its pungency.

Several convergent studies show that prolonged exposure to chemotherapy drugs may lead to the acquisition of drug-resistance in cancers,^136,137^ a major cause of mortality. As an example, small cell lung cancer (SCLC) initially responds very well to chemotherapy, with over 80% of patients showing remission. However, the cancer typically relapses within a few months and becomes unresponsive to chemotherapy/radiation.^138,139^ An exciting observation is that the region B capsaicin analogs augment the growth-suppressive activity of conventional chemotherapy and radiation therapy in lung cancers and oral cancers. Although these results are promising, a caveat is that the combinatorial growth-suppressive activity of capsaicin analogs and chemotherapy has only been studied in cell culture models. Such observations underscore the need for *in vivo* studies which will explore the “chemosensitization activity” of these region B capsaicin analogs.

Allosteric TRPV1 inhibitors represent a new class of region B capsaicin analogs,^140,141^ and an advantage of this class of compounds is that they do not block the ion channel function of TRPV receptors. Therefore, it may be envisaged that the administration of allosteric TRPV inhibitors will have fewer off-target effects compared to competitive TRPV1 inhibitors. Published reports have described the design and synthesis of several heterocyclic and peptidic allosteric TRPV1 ligands.^140,141^ Only one of these compounds, **7**, has been investigated for its growth-inhibitory activity in human breast cancer cell lines.^120^ It is hoped that other allosteric TRPV1 agonists will also be explored for their growth-suppressive activity in human cancers.

The growth-suppressive activity of region B capsaicin analogs has been predominantly demonstrated in cell culture systems and not in animal models. Such data underline the importance of examining the antineoplastic effects of region B capsaicin analogs *in vivo* in orthotopic and patient-derived xenograft (PDX) models. A key research area which needs to be explored is the pharmacokinetics of these region B capsaicin analogs^97,100,142^ since efficacy is dependent on its concentration at the target tissues. There is a paucity of studies exploring the pharmacokinetics of region B capsaicin analogs. The compounds **2** and **3** are the only two region B capsaicin analogs whose metabolism and biodistribution have been studied. The elucidation of pathways governing the metabolism of these compounds will pave the way to the designing of novel region B capsaicin analogs with greater stability and bioavailability *in vivo*. The region B capsaicin analogs **2** and **3** display potent antiangiogenic activity in cell culture and mice models.^105^ Similarly, the region B capsaicin analog **17** blocks the invasion of human prostate cancer cells.^122^ These data suggest that region B capsaicin analogs may suppress the distant metastasis of human cancers. We hope that future studies will shed light on the antimetastatic activity of region B capsaicin analogs.

The antineoplastic activity of region B capsaicin analogs is mediated via multiple signaling networks. An important question is whether the growth-inhibitory activity of these compounds require TRPV receptors or cannabinoid receptors or both of these receptors. Alternately, it may be possible that the growth-suppressive effects of these compounds are independent of both TRPV1 and cannabinoid receptors. The majority of research papers have examined the downstream effectors involved in the pro-apoptotic activity of the region B capsaicin analogs. It is hoped that future studies will shed light on the mechanisms by which these drugs bind to their cognate receptors on the cell membrane and how these ligand-bound receptors communicate to the cell-cycle machinery inside the nucleus. The development of novel antineoplastic region B capsaicin analogs with improved pharmacokinetic properties may revolutionize their applications in cancer therapy leading to new approaches to control cancer pain and block the growth and metastasis of cancers.

## Supporting Information Available

Three chemical synthesis routes to generate **5**: Figure S1 shows the structures of the reactants which were assembled together to obtain **5**, Scheme S1 outlines the free radical-based synthesis strategy to obtain **5** in a total of 41 steps, Scheme S2 describes an alternate route to synthesize **5** (comprising 27 steps) which contained an iridium(III) photocatalytic radical cyclization reaction, and Scheme S3 summarizes the most recent chemo-, regio-, and stereocontrolled assembly of **5** (in 15 steps) (PDF)

## Abbreviations Used

ADP: adenosine diphosphate
AST: aspartate aminotransferase
ALT: alanine aminotransferase
ATP: adenosine triphosphate
BAD: BCL2 associated agonist of cell death
Bcl-xL: B-cell lymphoma extra-large
BiP: binding immunoglobulin protein
CAS: capsiate
CI: combination index
CHO: Chinese hamster ovary
DBU: 1,8-diazabicyclo(5.4.0)undec-7-ene
DEAD: diethylazo-dicarboxylate
DH-CAS: dihydrocapsiate
CHOP: C/EBP homologous protein
DIBAL: di-isobutylaluminum hydride
DMAP: 4-dimethylaminopyridine
DMF: dimethylformamide
FAK: focal adhesion kinase
EGFR: epidermal growth factor receptor
Flk-1: fetal liver kinase 1
HCl: hydrochloric acid
HPE: high pressure extraction
HPLC: high pressure liquid chromatography
HUVEC: human umbilical cord endothelial cells
JNK: c-Jun N-terminal kinase
JAK: Janus kinase
KDR: kinase insert domain receptor
MAPK: mitogen-activated protein kinase
MAE: microwave-assisted extraction
MBq: mega becquerel
MPa: mega Pascal
MPLC: medium pressure liquid chromatography
MTT: 3-(4,5-dimethylthiazol-2-yl)-2,5-diphenyltetrazolium bromide
NADH: nicotinamide adenine dinucleotide (NAD) + hydrogen (H)
N-AVAM: N-acyl vanillyl acylamide
NDH-CAS: nordihydrocapsiate
NSG: NOD *scid* gamma;
NMR: nuclear magnetic resonance
NUCC: normal human urothelial cells
OSCC: oral squamous cell carcinoma
Pc: critical pressure
PDX: patient-derived xenograft
PKC: protein kinase C
PPA: polyphosphoric acid
ROS: reactive oxygen species
RTX: resiniferatoxin
ROPA: resiniferonol 9,13,14-orthophenylacetate
SAR: structure activity relationship
*Scid*: cevere combined immunodeficient
SCLC: small cell lung cancer
SFE: supercritical fluid extraction
SFE-HPE: supercritical fluid extraction combined with high pressure extraction
STAT3: signal transducer and activator of transcription 3
Tc: critical temperature
THF: tetrahydrofuran
TBDMSCl: tertiarybutyldimethyl silyl chloride
TLC: thin layer chromatography
TRAIL: TNF-related apoptosis-inducing ligand
TRPV: transient receptor potential vanilloid
UAE: ultrasound assisted extraction
UPLC: ultra performance liquid chromatography
UHP-HPLC: ultra high performance HPLC
US-SFE: ultrasound guided supercritical fluid extraction
VEGF: vascular endothelial growth factor
VE-cadherin: vascular endothelial cadherin
