# Supplementary material for: Anticancer Activity of Region B Capsaicin Analogs
Source: J Med Chem. 2023 Mar 31;66(7):4294–323. doi: 10.1021/acs.jmedchem.2c01594 (PMC10108357; doi:10.1021/acs.jmedchem.2c01594)
Supplement: Supplementary file 1 — jm2c01594_si_001.pdf [file jm2c01594_si_001.pdf]

SUPPORTING INFORMATION

Anti-cancer Activity of Region B Capsaicin Analogs

Kathleen C. Brown[1], Kushal J. Modi[1#], Reagan S. Light[1#], Ashley J Cox[1], Timothy E. Long[2], Rama S. Gadepalli[3], John M. Rimoldi[3], Sarah L Miles[1], Gary Rankin[1], Monica Valentovic[1], Krista L. Denning[4], Maria T. Tirona[5], Paul T. Finch[6], Joshua A. Hess[6] and Piyali Dasgupta[1*]

[1]Department of Biomedical Sciences, Toxicology Research Cluster, Joan C. Edwards School of Medicine, Marshall University, 1700 Third Avenue, Huntington, West Virginia, WV 25755; [2]Department of Pharmaceutical Sciences and Research, Marshall University School of Pharmacy, 1538 Charleston Ave, Huntington, WV 25701.[3]Department of Biomolecular Sciences, School of Pharmacy, Thad Cochran Research Center, University of Mississippi, University Ave, University, Mississippi, MS 38677; [4]Department of Pathology, Joan C. Edwards School of Medicine, Marshall University, 1400 Hal Greer Boulevard, Huntington, West Virginia, WV 25755, [5]Department of Hematology-Oncology, Edwards Cancer Center, Joan C. Edwards School of Medicine, Marshall University, 1400 Hal Greer Boulevard, Huntington, West Virginia, WV 25755; [6]Department of Oncology, Edwards Cancer Center, Joan C. Edwards School of Medicine, Marshall University, 1400 Hal Greer Boulevard, Huntington, West Virginia, WV 25755.

#These authors contributed equally to this work and should be considered equal second authors.

*Correspondence to: Piyali Dasgupta, Department of Biomedical Sciences, Joan C. Edwards School of Medicine, Marshall University, 1700 3rd Avenue, Huntington, WV 25755. E-mail: dasgupta@marshall.edu

Contents of Supplementary Information

Total number of pages =4 (S1-S5)

Total number of figures =1 (Figure S1)

Total number of reaction schemes=3 (Scheme S1-S3)

Supplementary Data, Scheme 1

Dr. Mayasaki Inoue's research group developed a novel free radical-based synthesis strategy to assemble the complex scaffold of **5**. The authors hypothesized that **5** could be obtained by the reaction of three distinct compounds, namely **43**, **44** and **45**[143] (Figure S1).

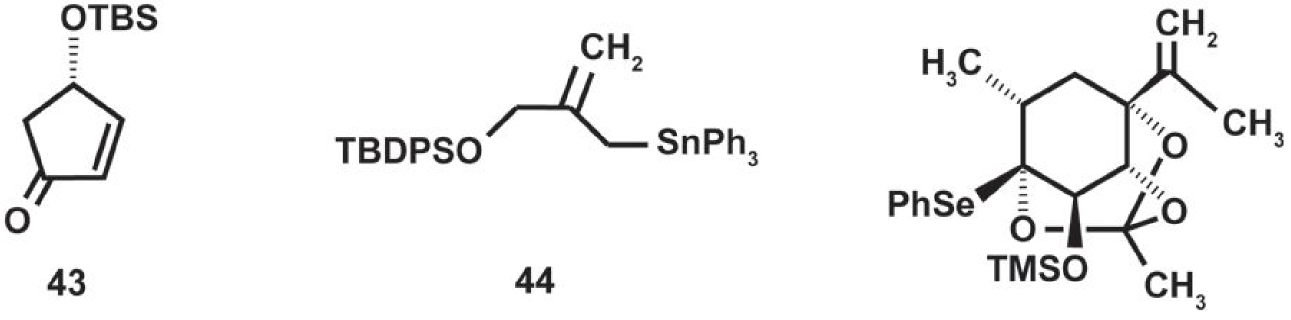

Figure S1. Structure of the reactants **43**, **44** and **45** for free radical based synthesis of **5**

This free radical-based synthesis strategy to obtain **5** comprised of a total of 41 steps and includes a three-component coupling reaction between the A-, C-rings, and a side-chain, followed by a 7-endo B-ring cyclization[143]. The compound **43** is commercially available in enantiomerically pure form. The starting reactant for **44** was 2-methyl-2-propen-1-ol **(Scheme S1)**. The compound **45** was chemically synthesized from the D-ribose derivative **46**. The free radical-based synthesis strategy to generate **5** is described in **Scheme S1**.

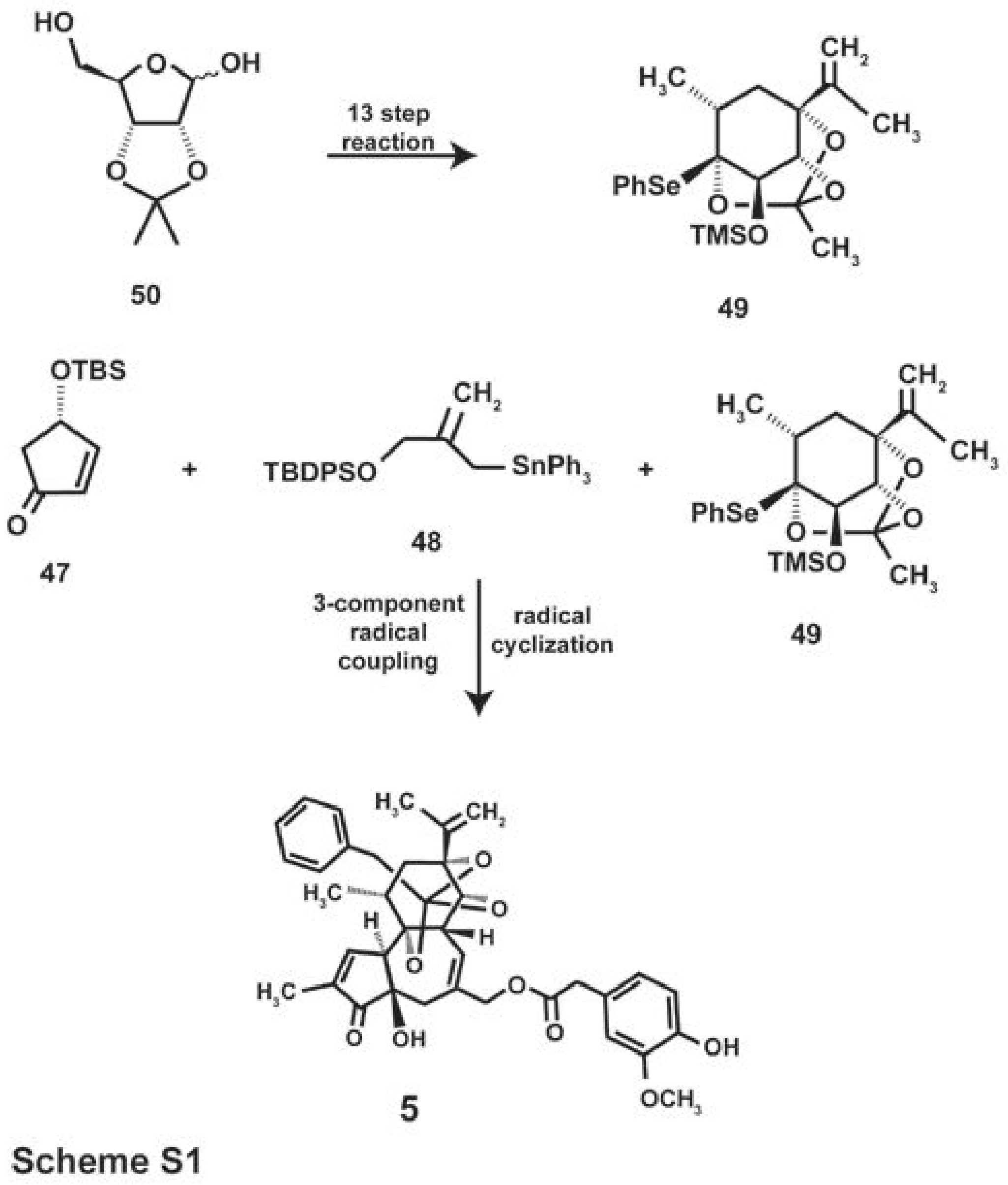

Scheme S1. Free radical reaction based total synthesis of 5

__Supplementary Data, Scheme 2__

In a subsequently published paper Inoue's research group improved the efficacy of the synthesis of **5** by using an iridium (III) photocatalytic radical cyclization (**Scheme S2**)[144]. This chemical

synthesis strategy to generate **5** used a D-ribose analog **47** as the starting material. This synthetic approach to obtain **5** contained 27 steps [144]. The yield of **5** was 40-fold higher than **Scheme 8** (in the main article) and **Supplementary Data, Scheme S1**.

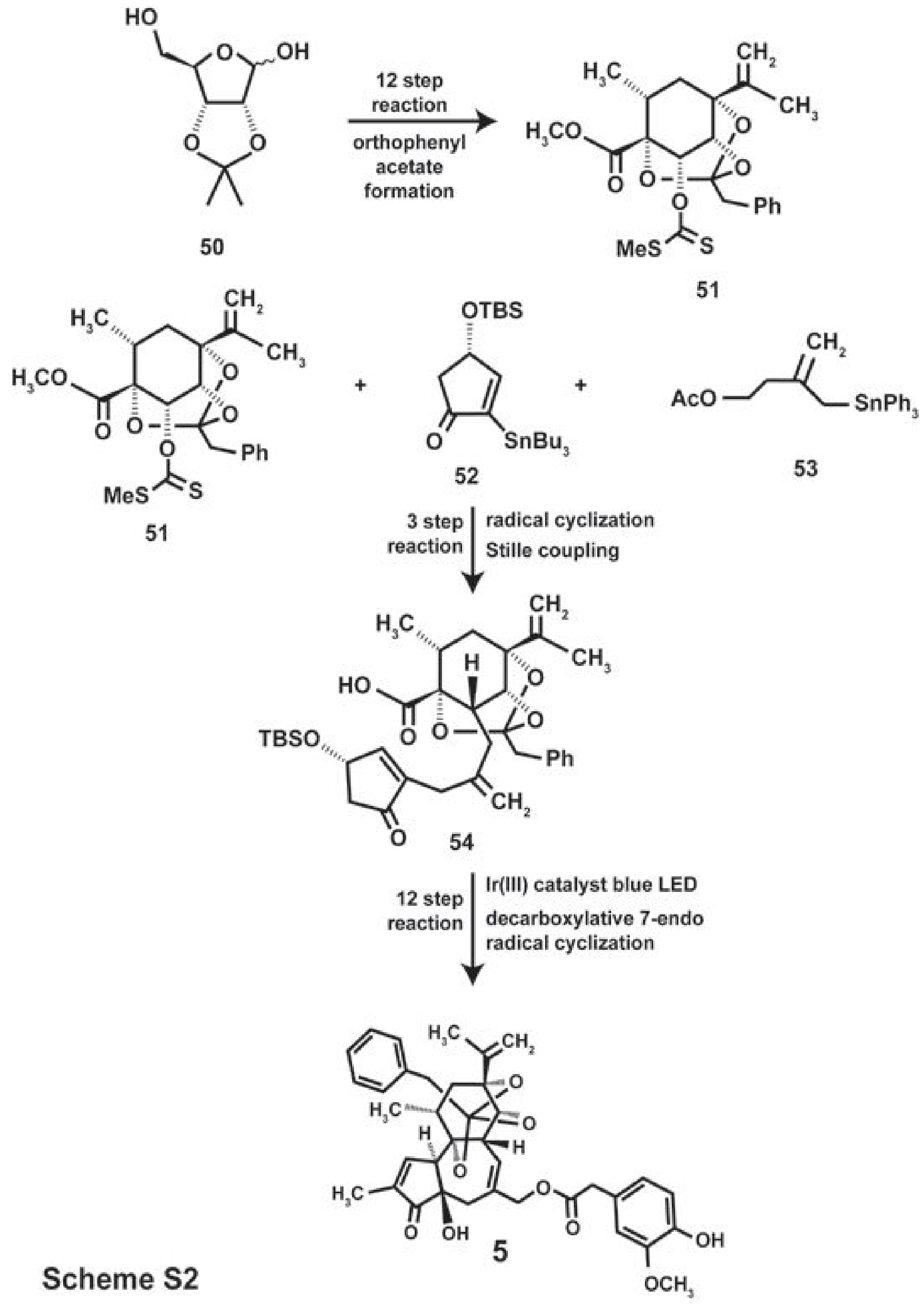

Scheme S2. Ir(III) photocatalyzed free radical based total synthesis of **5**

Supplementary Data, Scheme 3

The most recent synthesis accomplished by Dr. Thomas J. Maimone and his colleagues who completed a chemo-, regio-, and stereocontrolled assembly of **5** (**Scheme S3**) in 15 steps[145]. The commercially available (*Z*)-3-iodopropenoic acid methyl ester (**52**) was the starting material for the synthesis of **5**. A strength of this chemical synthesis protocol was that all the intermediate products in this fifteen-step synthesis method were obtained in good yields (≥ 40%) with a high degree of convergence and notable diastereocontrol **(Scheme S3)**.

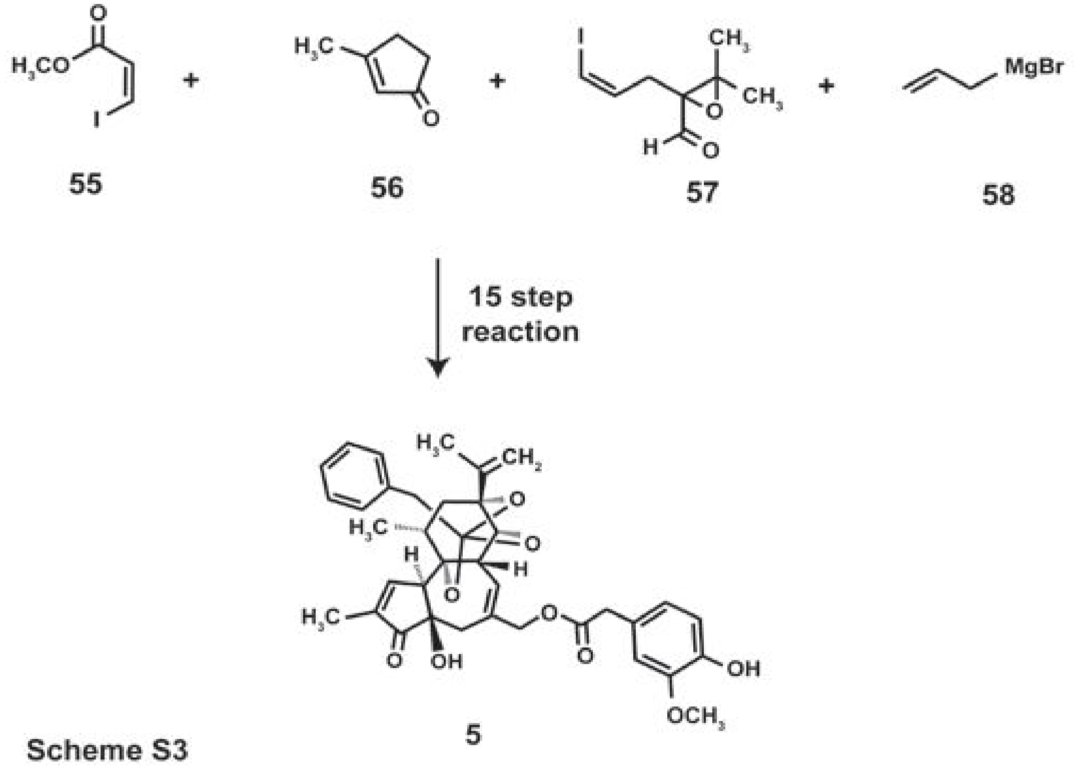

Scheme S3. A fifteen step total synthesis of **5**
